# Neutral interactions among non‐native plants are common

**DOI:** 10.1111/nph.71463

**Published:** 2026-07-24

**Authors:** Feng He, Zhiliang Yuan, Yumei Sun, Xincong Zhai, Xiangyu Tian, Yun Chen, Ragan M. Callaway, Xiao Sun

**Affiliations:** ^1^ State Key Laboratory of Crop Stress Adaptation and Improvement, School of Life Sciences Henan University Kaifeng 475004 China; ^2^ College of Life Sciences Henan Agricultural University Zhengzhou Henan 450046 China; ^3^ Division of Biological Sciences University of Montana Missoula 59812 USA

**Keywords:** co‐invasion, functional traits, invasional meltdown, invasive plants, phylogeny, plant–plant interactions, species richness

## Abstract

- Globalization has accelerated the establishment of non‐native plant species, increasing the prevalence of multispecies co‐invasions; yet interactions among co‐invaders remain poorly understood.
- We combined extensive field surveys of 3039 plots with a global meta‐analysis of 4415 observations from 122 experiments to quantify co‐invasion prevalence, its effects on non‐native plant performance, and the frequency of interaction types.
- Co‐invasion was common in the surveys, occurring in 40% of the plots. Meta‐analysis showed that co‐invasion reduced non‐native plant performance by 18% on average, indicating that invasional meltdown among plant species is not the norm. Most interactions were neutral (70%), whereas competition (18%) and facilitation (12%) were relatively uncommon. Thus, the widespread co‐occurrence of non‐native plants does not necessarily imply pervasive facilitation among co‐invaders. Crucially, the mean competitive outcome was context‐dependent. Interactions shifted from competition to facilitation with increasing non‐native richness and intensified competition with natives. Furthermore, phylogenetic distance among co‐invaders did not explain the interaction outcomes, whereas interactions among woody, nitrogen‐fixing species, or invaders with greater SLA produced mean neutral effects.
- These findings expand our understanding of the invasional meltdown hypothesis by showing that co‐invasion outcomes are context‐ and trait‐dependent and offer a predictive framework for assessing and mitigating multispecies invasion risks.

## Graphical abstract

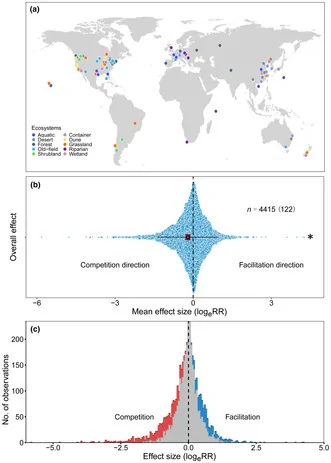

## 摘要

- 全球化加速了外来植物物种的引入与定殖，多种外来植物共同入侵现象愈发普遍。然而，共同入侵物种之间的相互作用仍缺乏系统认识。
- 我们结合3039个样地的大尺度野外调查和包含122个独立实验、4415个观测值的全球Meta分析，定量评估了共同入侵的发生频率和对外来植物表现的影响，以及不同相互作用类型的发生频率。
- 野外调查表明，约40%的样地存在两种及以上外来植物共同入侵。Meta分析发现，共同入侵平均降低外来植物表现18%，表明外来植物之间的入侵崩溃并非常态。大多数相互作用表现为中性(70%)，而竞争(18%)和促进(12%)相对较少。因此，外来植物的广泛共存并不意味着共同入侵物种之间普遍存在促进作用。更重要的是，共同入侵的平均相互作用具有明显的情境依赖性。随着外来植物丰富度增加，共同入侵物种之间的相互作用由竞争转向促进，同时对本地植物的竞争作用增强。此外，共同入侵物种之间的系统发育距离不能很好解释相互作用结果，而涉及木本植物、固氮植物或具有较高比叶面积的外来植物之间的相互作用，则总体表现为中性效应。
- 本研究拓展了对入侵崩溃假说的认识，表明共同入侵的结果受环境背景和物种功能性状共同调控，并为预测和管理多物种共同入侵风险提供了理论依据。

## Introduction

Globalization has accelerated the introduction and establishment of many non‐native plant species into new regions (van Kleunen *et al*., 2015; Seebens *et al*., 2020; Thakur *et al*., 2025). The ecological consequences of single‐species invasions are well documented, revealing profound impacts on native biodiversity and ecosystem functioning (Pyšek *et al*., 2012, 2020). However, multispecies co‐invasions, in which more than one non‐native species establishes concurrently, are common (Kuebbing *et al*., 2013; Brandt *et al*., 2023). The collective effects of these co‐invaders may just be additive, but they could also be synergistic and thus could lead to more harm to native ecosystems than would be predicted from single‐species invasions (Brandt *et al*., 2023; Ahmad *et al*., 2025). The disproportionate focus on pairwise interactions between non‐native and native species (Pyšek *et al*., 2012) leaves a knowledge gap: we know much less about the direction and strength of interactions among co‐invaders, and how these interactions may influence native plant communities. Exploring interactions among non‐natives is crucial for predicting future invasion risks.

Interactions among co‐invading plant species range from facilitation, which can generate synergistic effects, to competition, which may dampen the effects of individual invaders (Ahmad *et al*., 2025). Facilitative interactions have been studied in the context of the invasional meltdown hypothesis, which proposes that different non‐native organisms can amplify each other's survival, spread, and performance (Simberloff & Von Holle, 1999). By contrast, invasional interference describes scenarios where non‐native species inhibit one another, leading to reduced performance (Rauschert & Shea, 2012). The frequent co‐occurrence of non‐native species has motivated discussion of the invasional meltdown hypothesis (Kuebbing *et al*., 2015a; Braga *et al*., 2018). However, co‐occurrence alone does not demonstrate that interactions among co‐invaders are facilitative, because coexisting species may also experience neutral or competitive effects. A quantitative synthesis is therefore needed to identify the mean direction and strength of interactions among co‐invaders, as well as the frequencies of facilitation and competition in different experimental contexts and ecosystems.

The direction of interactions among co‐invaders may vary with environmental conditions, biological traits, species establishment order, coevolutionary history, and the protocols used to study them (Callaway & Walker, 1997; Kuebbing & Nuñez, 2016; Golivets & Wallin, 2018). First, facilitation tends to increase under more stressful abiotic conditions, such as in alpine and arid ecosystems, whereas competition predominates in more productive environments (Bertness & Callaway, 1994). Second, the biological traits of non‐native plant species (life histories, life forms, etc.) can have large effects on interactions (Callaway, 2007; Gómez‐Aparicio, 2009). For example, annual invaders often generate strong competitive interactions (Besaw *et al*., 2011), whereas perennial invaders may commonly facilitate each other (Thouvenot *et al*., 2013). Annual species interact with co‐occurring species over shorter temporal windows than perennials (Myster & Pickett, 1992). Similarly, herbs tend to exert stronger negative effects on co‐occurring species, whereas shrubs often exert facilitative effects (Gómez‐Aparicio, 2009). Nitrogen‐fixing invasive plants are also more likely to engage in facilitative interactions with other non‐fixing species (Carino & Daehler, 2002). However, non‐native plants with functional traits associated with high growth rates (such as specific leaf area and leaf N content) are more likely to suppress competitors and thus perform better (Feng & van Kleunen, 2016; Wang *et al*., 2023). Third, indirect interactions, those in which another species is involved, can be key drivers of co‐invasions (Kuebbing *et al*., 2015a; Chen & van Kleunen, 2022). Established invaders can facilitate subsequent non‐natives by modifying site characteristics, such as soil microbial communities and chemical properties (Flory & Bauer, 2014; Zhang *et al*., 2020). For example, the invasive plants *Rhamnus davurica* and *Lonicera maackii* perform better in soils pre‐conditioned by *Ligustrum sinense* than in uninvaded soils (Kuebbing *et al*., 2015a). Early‐arriving non‐natives may either inhibit or promote later arrivals by altering mutualist or pathogen accumulation, thereby modulating plant–soil feedbacks (Reinhart & Callaway, 2006; Inderjit & van der Putten, 2010).

Evolutionary relatedness and functional differences also play a critical role in shaping the direction and intensity of interactions among non‐native plants, as they influence functional traits and niche overlap (Martorell *et al*., 2021). Darwin proposed two opposing hypotheses to explain how coevolutionary history affects species interactions (Darwin, 1859; Bonanomi *et al*., 2011). Darwin's naturalization hypothesis suggests that closely related species tend to occupy similar niches, thereby intensifying the potential for competitive exclusion (Darwin, 1859). From functional trait perspectives, closely related species generally exhibit higher trait similarity, leading to overlapping resource use and thereby intensifying competition (Wagg *et al*., 2017). By contrast, the preadaptation hypothesis suggests that closely related species, sharing comparable traits and habitat preferences, may coexist more readily because they are pre‐adapted to similar environmental conditions (Cadotte *et al*., 2018). Species that are more distantly related often exhibit greater differences in functional traits; these differences may make it more likely for invasive species with stronger resource acquisition or utilization capabilities to emerge, thereby exerting stronger suppressive effects on competitors (Feng & van Kleunen, 2016). Both hypotheses have been supported by previous research. For example, invasion success can increase with greater similarity among non‐native species, potentially due to weaker competition among invaders compared to competition with native species, leading to indirect facilitative effects (Sheppard *et al*., 2018). Other studies have also found that *Erigeron canadensis* experienced stronger competition when growing with more distantly related non‐native species (Ren *et al*., 2023). Therefore, the role of phylogenetic and functional traits in mediating the direction and strength of interactions among non‐native plant species remains unclear.

To resolve contradictions in the literature and build a predictive understanding of co‐invasion, we integrated a large‐scale field survey with a global meta‐analysis. Field surveys quantified the prevalence of co‐occurring non‐native plants at invaded sites, thereby providing the ecological context for interactions among co‐invaders. However, co‐occurrence alone cannot reveal whether these species facilitate, compete with, or have little effect on one another. We therefore conducted a global meta‐analysis to determine the mean direction and strength of interactions among co‐invaders and to evaluate how these interactions vary with experimental conditions, functional traits, species richness, and phylogenetic relatedness. We aimed to answer the following questions: (1) What is the mean outcome of interactions when non‐native plant species co‐invade? (2) How common and intense is facilitation in co‐invasions, that is, invasional meltdown? (3) Do experimental conditions, increasing invader richness, phylogenetic and functional attributes, alter the strength and direction of these interactions? In addition, to link interactions among non‐native species to their potential ecological implications, we used available data to examine the responses of co‐occurring native plant species to co‐invasion. Together, these complementary approaches link the prevalence of co‐invasion in natural communities with the mechanisms governing interactions among co‐invaders, providing a mechanistic basis for predicting the ecological consequences of multispecies invasions.

## Materials and Methods

### Field survey on co‐invasion prevalence

**Fig. 1:**
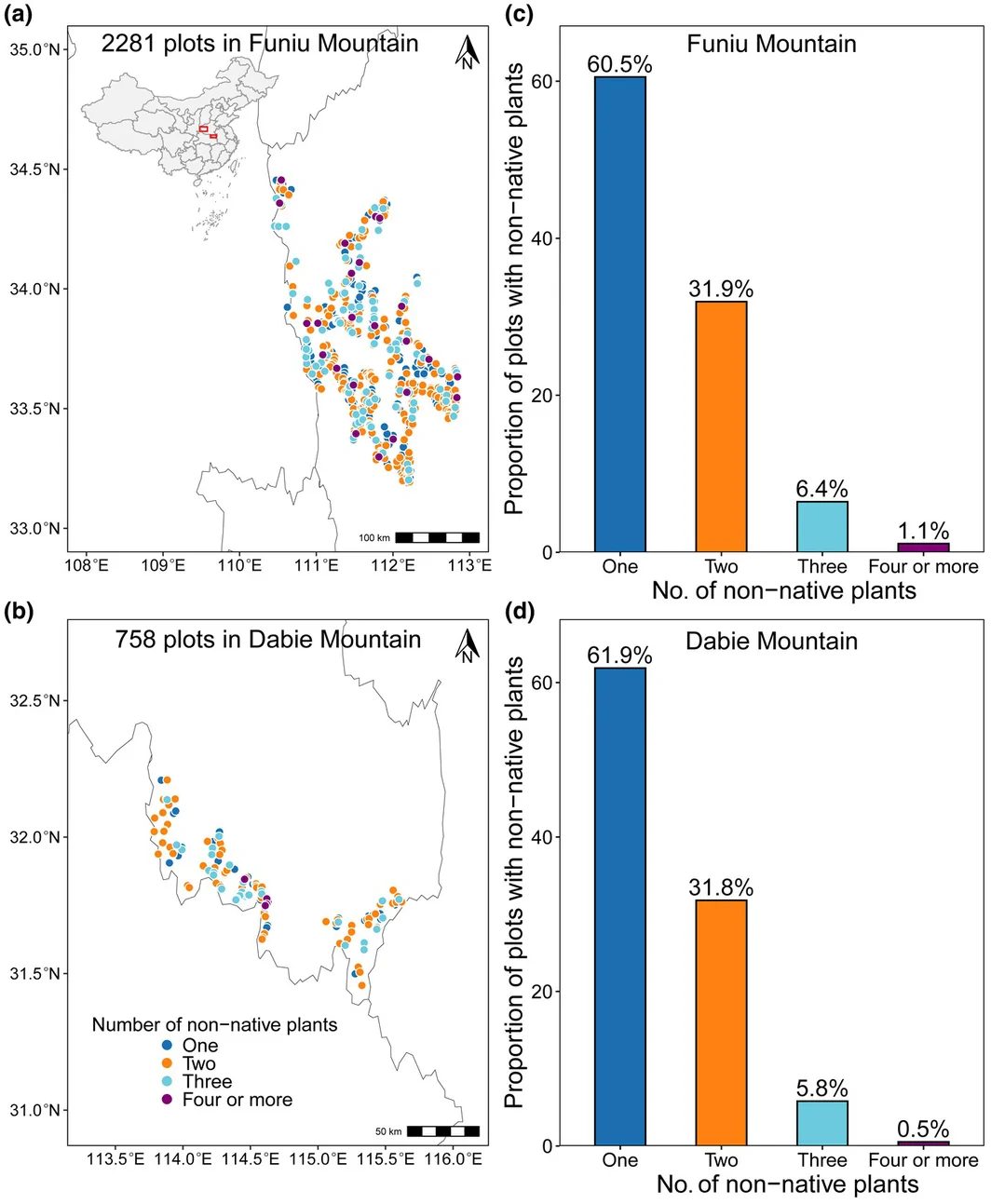
Spatial distribution and richness of co‐occurring non‐native plant species across 3039 survey plots. Geographic distribution of sampling plots in the Funiu Mountains (a) and Dabie Mountains (b), with colors indicating non‐native plant richness. Frequency distributions of non‐native plant richness in the Funiu Mountains (c) and Dabie Mountains (d). Values above bars represent the proportion of plots in each richness category.

To document patterns of non‐native plant co‐occurrence, we conducted systematic field surveys across the Dabie Mountains and Funiu Mountains in central China and recorded instances of co‐occurrence in plots containing non‐native plants (Fig. 1a,b). We surveyed four common habitat types that represent major anthropogenic and semi‐natural environments in the region: desert, roadside, cropland, and riparian. For each type, we first established at least five independent sites, each covering 1 km^2^. To adequately capture within‐community heterogeneity, we haphazardly established a minimum of five 1 m × 1 m plots within each site, arranging them in either a cross or zigzag pattern. To minimize spatial autocorrelation, sites were spaced at least 1 km apart. Plant species recorded during the field surveys were classified as either invasive alien or native to China based on information from the Chinese Virtual Herbarium (CVH; http://www.cvh.ac.cn/) and *The Checklist of the Alien Invasive Plants in China* (Ma & Li, 2018). All surveys were conducted by the same trained research team between April and October 2023, covering the main growing season in the study region and ensuring consistency among observations. We surveyed a total of 3039 sample plots, including 2281 plots in the Funiu Mountains and 758 plots in the Dabie Mountains.

### Literature search and data compilation

To quantitatively assess interactions among non‐native plants during co‐invasion, we conducted a systematic literature search to build a global dataset following the PRISMA guidelines (Supporting Information Fig. S1) (O'Dea *et al*., 2021). We systematically searched the ISI Web of Science, Scopus, and China National Knowledge Infrastructure (CNKI) databases without publication year restrictions, using the following search terms: (invasional meltdown OR multiple invasive OR synergistic invasion OR co‐invasion OR co‐occur* OR secondary invasion OR invasional interference) AND (invas* OR non‐native OR non‐indigenous OR alien OR exotic) AND (plant*). We supplemented this search by examining the reference lists of previous meta‐analyses and key review articles on the topic (Kuebbing & Nuñez, 2015, 2016; Braga *et al*., 2018; Golivets & Wallin, 2018; Ahmad *et al*., 2025). We conducted iterative literature searches until January 2025, yielding a total of 13 701 publications.

Studies were included in our meta‐analysis based on the following pre‐defined criteria:

1. The study investigated interactions between two or more non‐native plant species, including either direct interactions or indirect interactions, with effects that could be isolated from other treatments;
2. Studies had to include both a treatment in which non‐native plant species interacted and a control in which they did not. Direct interactions included additive designs, replacement designs, removal experiments, non‐native richness gradient experiments, and naturally occurring contrasts in the presence vs absence of non‐native neighbors in field surveys. Indirect interactions were limited to plant–soil feedback experiments, where non‐native plant species conditioned the soil, which was then used to grow different subsequent non‐native plant species (see Methods S1 for detailed experimental designs of interactions);
3. Studies provided quantitative data on at least the germination, growth, reproduction, or community‐level variables of non‐native species under different treatments. For germination variables, germination rate (i.e. proportion of germinated seeds) was measured. For growth variables, measurements included total biomass or biomass of different plant parts (leaves, stems, shoots, and roots), length of roots and shoots, leaf area and number, number of tillers, stem diameter, survival, and mortality. For reproductive variables, measurements included the biomass of seeds, fruits and flowers, and the number of seeds, fruits, flowers and ramets. For community variables, the proportion of biomass, cover, richness and abundance of non‐native plants were included; and
4. Studies were required to provide the mean values, statistical variances (SD, SE, or 95% confidence intervals), and sample sizes.

A total of 117 studies met all criteria (see Supporting Information). We extracted data (means, statistical variances and sample sizes) either directly from text, tables, or raw data, or by digitizing figures using Getdata Graph Digitizer (v.2.26, Russian Federation). For studies with multiple time points, we retained only the final measurement to avoid temporal autocorrelation. In studies involving pairwise interactions, the effect of non‐native plant A (neighboring plant) on non‐native plant B (focal plant) vs the effect of plant B on A was distinguished as two observations. If multispecies experiments provided only aggregated values for multiple non‐native plants without allowing for the identification of individual species, or if the report only presents the average performance of multiple non‐native plants, we retained the data at the experimental level. In such cases, we assigned unique identifiers to distinguish different experiments. These identifiers represent different experiments rather than individual species. Information on whether a species is non‐native or native was extracted from the study where available. If the study did not report whether the species was non‐native, we used the Global Naturalized Alien Flora database (van Kleunen *et al*., 2019), the Germplasm Resources Information Network (USDA; https://www.ars‐grin.gov/), and the Plants of the World Online (http://powo.science.kew.org/) to extract the non‐native status of the species.

To explore the factors influencing non‐native plant species performance under co‐invasion, we extracted metadata on geographic location, ecosystem type, experiment duration, experimental type, experimental design, the number of non‐native species involved, species identity and biological traits (life histories, life forms, and nitrogen‐fixing status), relative planting time of the focal non‐native plant compared to the neighboring non‐native plant (early, simultaneous, or late planting). We also recorded whether the study was a removal or a plant–soil feedback experiment. Study types were classified as glasshouse experiments, field experiments, or correlative spatial associations. Glasshouse experiments tested interactions between non‐native species in controlled environments. Field experiments implemented the addition, replacement, or removal designs. Spatial associations describe observational comparisons in natural sites, specifically studies examining the performance of non‐native plants where a focal non‐native species occurred either alone or together with other non‐native species. We obtained information on the life histories and life forms of non‐native plants from articles and the TRY plant trait database (Kattge *et al*., 2020). If trait information was unavailable from these sources, we consulted additional published references and authoritative online sources. When species identity or trait information remained unavailable, we excluded these observations from trait‐based analyses. Life histories were classified as short‐lived (annual and biennial) or long‐lived (perennial). Plant life forms were categorized as forbs, grasses, or woody species. In addition, we extracted the performance of native plant species in monocultures vs mixed non‐native invasion treatments to assess impacts on native communities (see Methods S1 for details on whether data on native plants were extracted).

### Phylogeny and functional traits

To quantify the phylogenetic relationships among the non‐native plants in our database, we constructed a phylogenetic tree encompassing all 202 species. We standardized the scientific names of the species and retrieved their genus and family information using the taxize package, which accesses taxonomic data from the National Center for Biotechnology Information (NCBI) database (Chamberlain *et al*., 2020). We then built a phylogenetic tree for all non‐native plants using the v.phylomaker2 package, which generates phylogenies based on molecular data from GenBank and is anchored to the backbone of the Open Tree of Life (Jin & Qian, 2022). Phylogenetic relatedness among non‐native plants was quantified using Faith's index, which captures the total evolutionary history represented by the set of species involved in an interaction. Faith's index was calculated as the sum of total branch lengths connecting all non‐native species included in each observation, based on the constructed phylogenetic tree (Faith, 1992; Chen *et al*., 2025).

To examine how plant functional traits influence interactions among non‐native plants, we extracted species trait data, including specific leaf area (SLA), leaf nitrogen content (leaf N), and leaf phosphorus content (leaf P), from the TRY Plant Trait Database (Kattge *et al*., 2020). These traits are widely used in ecology research and reflect the plant economic spectrum (Díaz *et al*., 2016). Because trait data availability varied among species, including multiple traits simultaneously would substantially reduce the dataset. Therefore, we focused on these core traits and calculated functional diversity separately for each trait. Functional diversity was calculated using functional dispersion (FDis), based on the subset of focal and neighboring non‐native plants for which trait data were available (Laliberté & Legendre, 2010). FDis quantifies the mean dispersion of species to the centroid in trait space, weighted by species planting proportion, and was calculated using the fd package (Laliberté & Legendre, 2010).

### Effect size calculation

We calculated the effect size of co‐invasion on non‐native species performance using the natural logarithm of the response ratio (log_e_RR), calculated after (Borenstein *et al*., 2009):

(Eqn 1) $$\log_{e}\mathrm{RR}=\log_{e}\left(\bar{X_{\mathrm{mix}}}/\bar{X_{\text{mono}}}\right)$$

where $\bar{X_{\mathrm{mix}}}$ is the mean performance of non‐native species in co‐invasion (growing with other non‐native species), and $\bar{X_{\text{mono}}}$ is the mean performance under single‐species invasion (growing alone or with the same non‐native species). To ensure consistency in the direction of effect sizes, we reversed the sign for traits where higher values indicated lower plant performance (e.g. high mortality). Thus, negative log_e_RR values correspond to cases where co‐invasion reduced non‐native plant performance, whereas positive values represent a performance increase. The corresponding sampling variance of log_e_RR was calculated as follows (Borenstein *et al*., 2009):

(Eqn 2) $$\nu =\frac{S_{\mathrm{mix}}^{2}}{n_{\mathrm{mix}}\times {\bar{X_{\mathrm{mix}}}}^{2}}+\frac{S_{\text{mono}}^{2}}{n_{\text{mono}}\times {\bar{X_{\text{mono}}}}^{2}}$$

where *S* denotes the SD, *n* denotes the sample size, and the subscripts ‘mix’ and ‘mono’ refer to co‐invasion and single‐species invasion treatments, respectively. When data on native plants were available, the same formulas were used to calculate the effect size and sampling variance for native plants (Eqns 1, 2). In this case, log_e_RR quantifies the response of native plants to co‐invasion, defined as the performance of native plants when growing with multiple non‐native plant species relative to their performance when growing with a single non‐native neighbor. Therefore, this calculation method can show the performance changes of native plants as a single‐species transition to a co‐invasion scenario.

From the effect size variance (*v*), the SE and confidence intervals for each value of the effect size (i.e. log_e_RR) can be calculated as:

(Eqn 3) $$\mathrm{SE}=\sqrt{\nu }$$

(Eqn 4) $${\mathrm{CI}}_{95\%}=1.96\times \mathrm{SE}$$

Once the interaction effect sizes of non‐native plant species in co‐invasion were calculated, these interactions were categorized into competitive, neutral, and facilitative outcomes. Neutral interactions occurred when plant species had no effect on each other and were defined as no significant difference in the performance of non‐native plants between co‐invasion and single invasion scenarios, with the 95% confidence interval (CI) overlapping zero. Competitive interactions were indicated by reduced performance of non‐native plants in co‐invasion, where the upper bound of the 95% CI was below zero. Facilitative interactions were indicated by improved performance of non‐native plants in co‐invasion, with the lower bound of the 95% CI above zero.

### Statistical analyses

To analyze the field survey data, we constructed a species‐by‐site matrix of non‐native plant occurrences across all sampled plots. Non‐native plant richness was calculated as the total number of non‐native species recorded per plot. We analyzed the occurrence frequency of non‐native plant richness across all plots in the Funiu Mountains and the Dabie Mountains independently.

To complement the field surveys and evaluate general interaction outcomes under co‐invasion, we conducted a meta‐analysis of non‐native plant performance under co‐invasion using weighted mixed‐effects models implemented in the *rma.mv* function of the metafor package (Viechtbauer, 2010). The model incorporated several random effects: (1) study ID, to account for between‐study variation and quantify between‐study heterogeneity; (2) individual effect size ID, to account for within‐study non‐independence and quantify within‐study heterogeneity; and the identities of (3) the focal and (4) the neighboring non‐native plant species, to account for species‐specific effects. The weight for each observation was calculated by the inverse of its sampling variance.

First, we fitted an intercept‐only multilevel random effects model to estimate the overall mean effect of co‐invasion on non‐native species performance. In this model, we used effect size (log_e_RR) as the response variable and estimated total heterogeneity and heterogeneity associated with each random effect using the *i2_ml* function from the orchard package (Nakagawa *et al*., 2023). Second, to explore the sources of heterogeneity, we fitted multi‐level mixed‐effects models (i.e. meta‐regression models) to assess the contribution of various moderators (fixed effects) in explaining heterogeneity in effect sizes. These moderators included: response type, ecosystem type, experiment type, experiment design, whether the study involved a plant–soil feedback experiment, whether it was a removal experiment, planting time of the focal non‐native plant species, life histories, life forms, nitrogen‐fixing ability, and functional traits of both focal and neighbor non‐native species, the number of non‐native species, functional diversity, and phylogenetic distance among the non‐native species. We further assessed the robustness of these factors by fitting full models that simultaneously incorporated multiple moderators while controlling for other factors. However, because experimental context variables and functional trait variables differed in data completeness and sample coverage, we fitted separate models for these two sets of predictors. Third, to test whether the impact of co‐invasion on native plant species is related to the performance and numbers of non‐native species, we fitted a separate meta‐regression model using the effect size for native species as the response variable and both the effect size and numbers of non‐native species as moderators. We calculated 95% CI for each mean effect size to assess statistical significance. The effect size was considered statistically significant when its CIs did not overlap zero. To interpret the influence of co‐invasion on non‐native species performance on the raw data scale, we back‐transformed the natural log response ratio (log_e_RR) using the following formula: $e^{\log_{e}\mathrm{RR}}-1$. For example, an log_e_RR of −0.6 indicates a 45.1% decrease in non‐native species performance under co‐invasion, whereas an log_e_RR of 0.4 indicates a 49.2% increase.

To evaluate shifts in the proportion of interactions among non‐native plant species, a vote‐counting method was conducted after classifying the interactions. Differences in the frequencies of interaction categories across moderators were tested using Fisher's exact tests. To further examine how moderator variables influence the relative probabilities of interaction outcomes, we fitted a Bayesian multinomial logistic regression model using the brms package (Bürkner, 2017), with interaction category as the categorical response and moderator type as the predictor. The model was estimated using four independent chains, each comprising 4000 iterations. Half of these iterations were designated as warm‐up, so there was a total of 8000 iterations to estimate the posterior distribution of each parameter.

We assessed the potential for publication bias using three complementary approaches. First, we visually inspected funnel plots for asymmetry. Second, we conducted a modified Egger's regression test by incorporating the square root of the sampling variance (i.e. SE) as a moderator in a meta‐regression model (Viechtbauer & Cheung, 2010). Finally, we evaluated the temporal trend in effect sizes. Time‐lag bias occurs when earlier published studies yield larger effects than later ones (Trikalinos & Ioannidis, 2005). Therefore, we constructed a meta‐regression model with publication year as the moderator variable and effect size as the response variable to test for the presence of time‐lag bias. When publication bias was detected, we used Rosenberg's fail‐safe number (Rosenberg's *N*) to evaluate whether the potential influence of unpublished studies could alter our overall conclusions (Koricheva *et al*., 2013).

## Results

### Prevalence of co‐invasion in the field

Field surveys in the Funiu and Dabie Mountains quantified the occurrence patterns of non‐native plant species at invaded sites. A total of 1189 plots (39.1%) were co‐invaded by two or more non‐native plant species (Fig. 1a,b). The distribution of non‐native species richness was similar between regions: 39.5% of plots in the Funiu Mountains and 38.1% in the Dabie Mountains contained multiple non‐native species, whereas 60.5% and 61.9% of plots, respectively, contained only a single non‐native species (Fig. 1c,d).

### Dataset characteristics and global representation

**Fig. 2:**
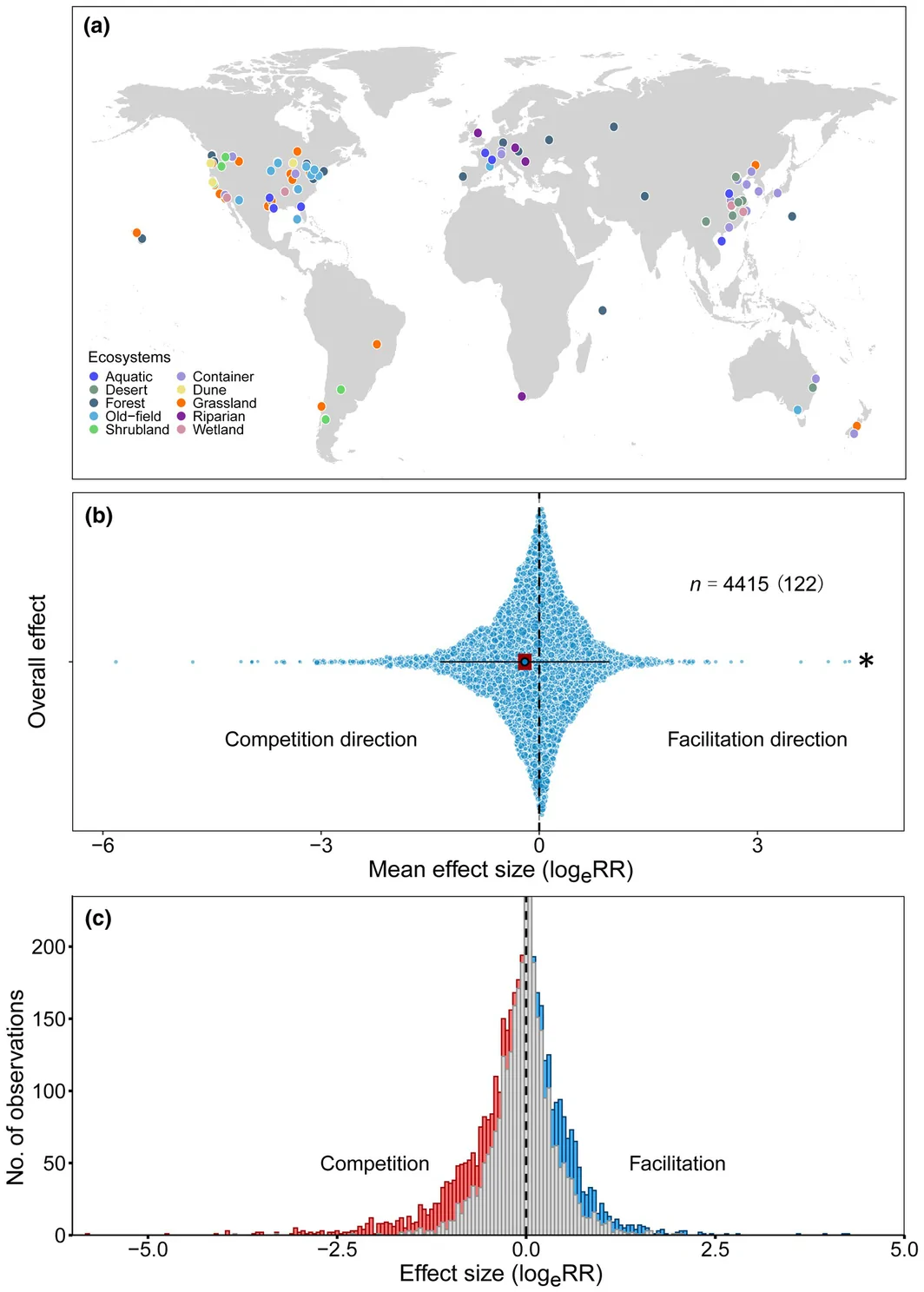
Global distribution of studies and overall effect of co‐invasion for all metrics of non‐native plant performance combined. (a) World map showing geographic origins of studies included in the meta‐analysis, colored by ecosystem type. (b) Orchard plots showing the overall effect size of co‐invasion on non‐native plant performance. Negative mean effect sizes (log_e_RR) reflect net competitive interactions among co‐occurring non‐native plants, whereas positive values reflect net facilitative interactions. Points represent estimated means, while thick red whiskers indicate the 95% confidence intervals (95% CI) and thin black whiskers show 95% prediction intervals. Asterisks indicate estimates whose 95% CI does not overlap zero. (c) Histogram of the distribution of effect sizes (log_e_RR) for non‐native plant performance across all observations; *y*‐axis shows the number of effect sizes per bin. The dashed line represents the zero line. Red‐ and blue‐shaded areas indicate significant competitive and facilitative interactions, respectively, whereas the gray‐shaded area represents non‐significant (neutral) interactions. *n* represents the number of observations and experiments.

The meta‐analysis synthesized a global dataset of 4415 observations extracted from 117 studies, comprising 122 independent experiments. The studies were primarily from North America (818 observations from 60 experiments) and Europe (2844 observations from 15 experiments), with contributions from Asia (583 observations from 35 experiments) and other continents (Table S1). Most observations were from forests (474 observations from 22 experiments) and grassland (143 observations from 23 experiments) (Fig. 2a; Table S2). Glasshouse experiments formed the bulk of the experimental data (3778 observations from 65 experiments), complemented by field experiments (544 observations from 47 experiments), and spatial associations (93 observations from 16 experiments) (Table S3). Replacement designs included 3640 observations from 51 experiments, while addition designs included 326 observations from 31 experiments (Table S4). Studies involving two non‐native plants were the most common (3845 observations from 75 experiments), while studies involving three or more were rare (139 observations from 14 experiments) (Table S5). The dataset included 16 field removal experiments and 15 plant–soil feedback experiments involving non‐native plants (Tables S6, S7). Growth‐related metrics were most frequently reported (3169 observations from 117 experiments), followed by reproduction (1156 observations from 22 experiments) and germination (90 observations from 9 experiments) (Table S8).

### Overall effects of co‐invasion on non‐native plant performance

The meta‐analysis demonstrated that, considering all metrics combined, co‐invasion reduced the performance of non‐native plants by an average of 18.0% compared to single‐species invasion scenarios (log_e_RR = −0.198, 95% CI = [−0.291, −0.105]; Fig. 2b). However, the direction of interactions varied substantially, with 69.9% of observations indicating neutrality (3088 observations) or no effect of co‐invaders on each other, 18.3% showing competition (807 observations), and 11.8% showing facilitation (520 observations) interactions among non‐native plants (Fig. 2c; Table S9). Effect sizes showed high heterogeneity (*I* ^2^ = 96.2%), with between‐study (*I* ^2^ = 35.9%), within‐study (*I* ^2^ = 30.0%), between‐focal‐plant (*I* ^2^ = 13.9%), and between‐neighboring‐plant (*I* ^2^ = 16.4%).

**Fig. 3:**
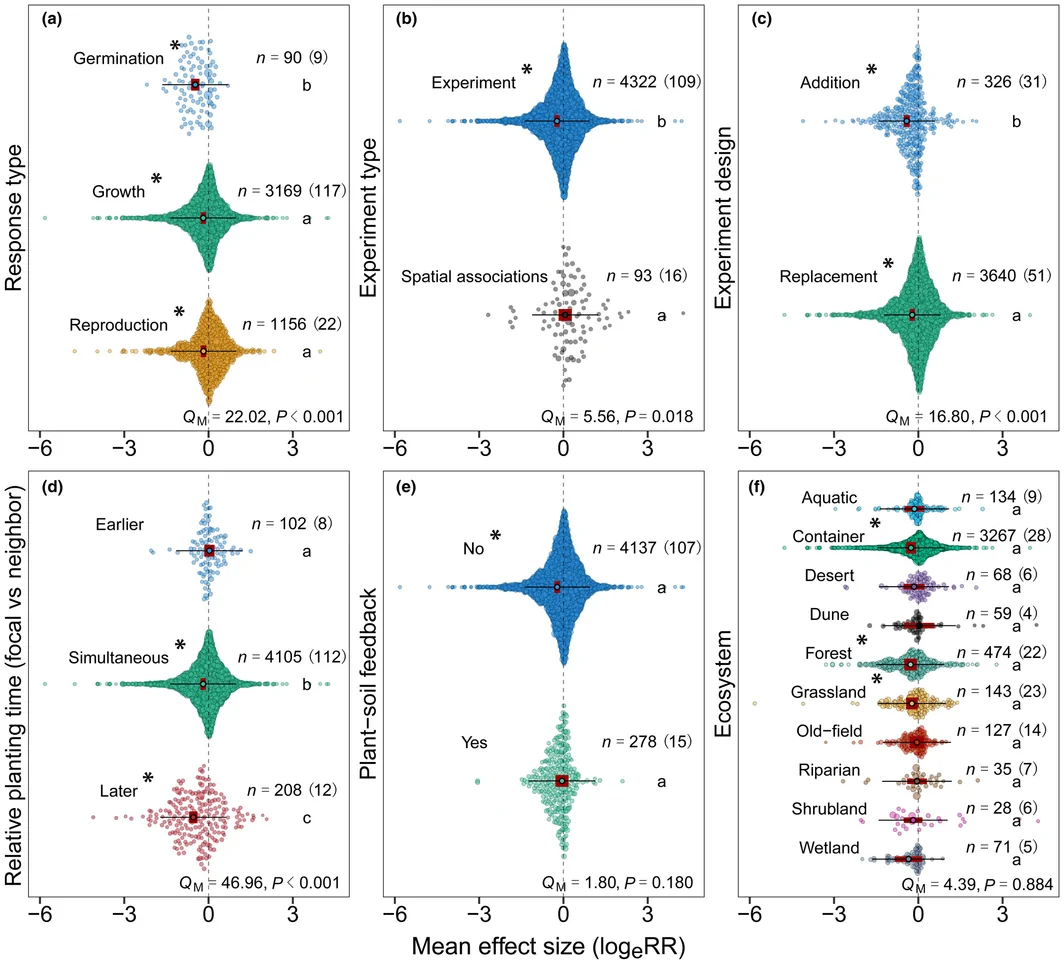
Effects of experimental context and ecosystem type on non‐native plant performance under co‐invasion. Study design factors include response type (a), experimental type (b), experimental design (c), planting sequence (d), plant–soil feedback experiments (e), and ecosystem (f). Points represent estimated means, while thick red whiskers indicate the 95% confidence intervals and thin black whiskers show 95% prediction intervals from multilevel meta‐regression models. Asterisks denote significant effects. *n* represents the number of observations and experiments. The *Q*_M_ and *P*‐value are presented in each subplot. *P* < 0.05 indicates a significant difference between different factor levels and is marked with different letters.

The mean negative effects of co‐invasion were consistent across different performance metrics, with significant suppression for germination, growth, and reproduction (Fig. 3a; Table S10). The proportion of interactions among non‐native plants varied significantly across different plant performance metrics (*P* < 0.001), with the majority of interactions being neutral (Fig. S2a; Table S11).

### Context‐dependent interactions among co‐invading non‐native plants

The magnitude of co‐invasion effects varied substantially with experimental context. Manipulative experiments (glasshouse and field) yielded the strongest competitive interactions, reducing focal plant performance by 20.6% (Fig. 3b; Table S10). Spatial association analyses had a non‐significant yet slightly positive trend and higher performance than manipulative experiments (+34.2%; Fig. 3b). The proportion of interactions among non‐native species differed significantly between spatial association and manipulative experiments (*P* < 0.001); spatial associations (36.6% of the observations) were 3.3 times more likely to indicate facilitation than manipulative experiments (11.2% of the observations) (95% CI [1.000, 1.896]; Fig. S2b; Table S11). In addition, both additive and replacement designs showed significant negative effects, but competition was weaker in replacement designs (Fig. 3c; Table S10). Replacement designs had a higher frequency of facilitative (12.0% of the observations; 95% CI [0.961, 2.534]) and lower frequency of competitive (16.7% of the observations; 95% CI [−1.065, −0.565]) interactions compared to additive designs (competition: 34.0%, facilitation: 2.2%) (Fig. S2c; Table S11).

In the field, removal experiments did not significantly affect focal species performance or interaction proportions (Fig. S3a,b; Tables S10, S11). When focal and neighboring non‐native plants were planted simultaneously or later, the interactions were competitive, with stronger competition for later arrivals (Fig. 3d; Table S10). Focal non‐native species planted earlier were not affected by neighbors and performed better than those planted simultaneously (+25.0%; Fig. 3d; Table S10). Competitive interactions occurred more frequently when species were planted simultaneously (17.9%; 95% CI [0.801, 2.685]) or later (33.2%; 95% CI [1.467, 3.457]) than earlier (4.9%) (Fig. S2d; Table S11).

Non‐native plants grown in soil conditioned by heterospecific non‐native plants were not affected, but those grown together with heterospecific non‐native plants were suppressed (Fig. 3e; Table S10). Plant–soil feedback altered interaction types (*P* = 0.015) by reducing the frequency of facilitation (6.8%; 95% CI [−1.145, −0.157]) relative to no‐feedback (12.1%) (Fig. S2e; Table S11).

Across ecosystems, competitive interactions were observed in forest and grassland, particularly in growth (Figs 3f, S4b; Table S10). However, neutral interactions were observed in aquatic, desert, dune, old‐field, riparian, shrubland, and wetland systems (Fig. 3f; Table S10). In all systems except shrublands, neutral interactions exceeded 50% (Fig. S2f). Importantly, in aquatic, dune, forest, and grassland, competition occurred more frequently than facilitation, while facilitation only exceeded competition in desert (Fig. S2f; Table S11). In old‐field, riparian, and shrubland, there was no difference in the frequency of competition and facilitation, whereas in wetlands, all significant interactions were competitive interactions (43.7%) (Fig. S2f; Table S11). Furthermore, experiment duration had relatively weak effects (Fig. S5). We also examined the effects of different experimental contexts on the performance of non‐native plants under co‐invasion, while accounting for other factors by fitting them into one model, and the results were qualitatively consistent with those obtained from separate analyses (Table S12).

### Trait‐mediated interactions among non‐native plant invaders

**Fig. 4:**
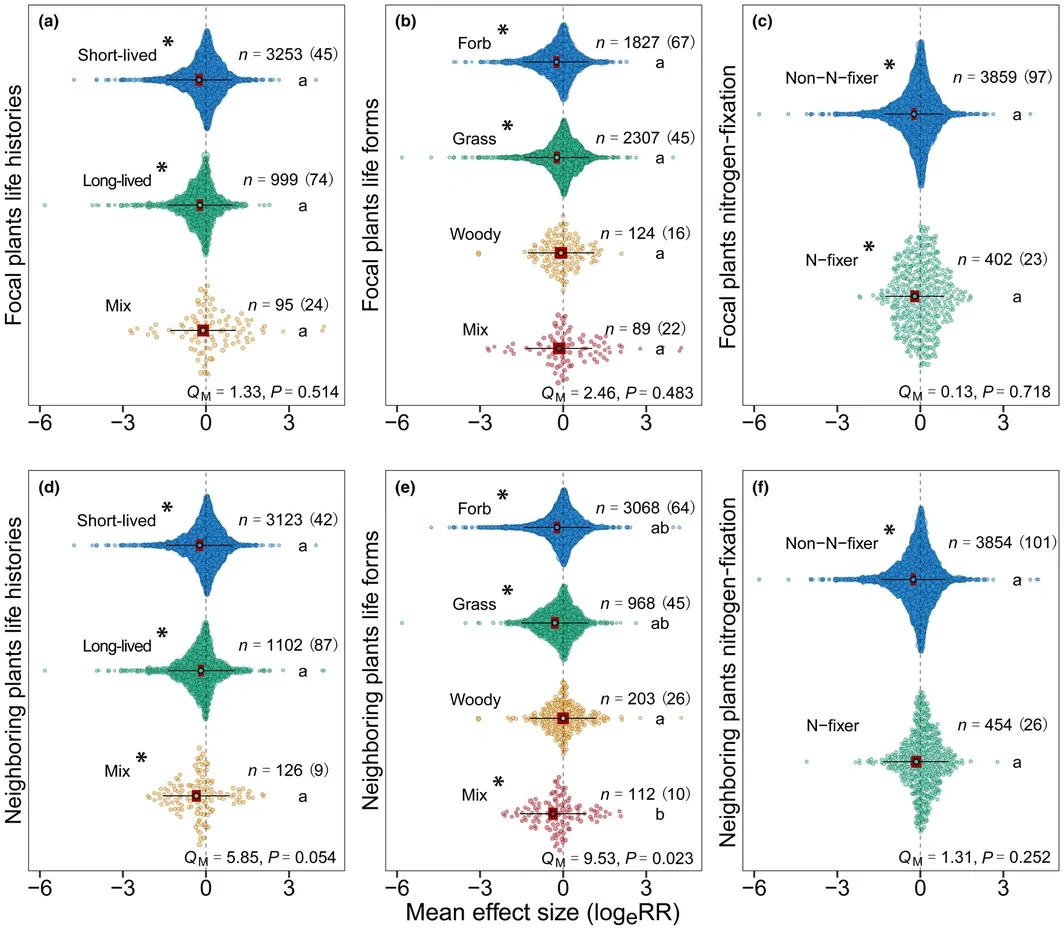
Modulation of co‐invasion effects by biological traits of focal and neighboring plants. Orchard plots showing the effects of biological traits of focal plants (a–c) and neighboring plants (d–f) on co‐invasion outcomes. Traits include life histories (a, d), life forms (b, e), and nitrogen‐fixing capacity (c, f). Points represent estimated means, while thick red whiskers indicate the 95% confidence intervals and thin black whiskers show 95% prediction intervals from multilevel meta‐regression models. Asterisks denote significant effects. *n* represents the number of observations and experiments. The *Q*_M_ and *P*‐value are presented in each subplot. *P* < 0.05 indicates a significant difference between different factor levels and is marked with different letters.

Co‐invasion reduced the performance of both short‐ and long‐lived species, particularly when measured as growth (Figs 4a,d, S6a–c, S7a–c; Tables S13, S14). There was no difference in mean interaction outcomes between life histories, whether they were the focal or neighbor species (Fig. 4a,d). Short‐ (focal: 14.5% vs 12.2%, neighbor: 13.8% vs 11.9%) and long‐lived (focal: 31.1% vs 9.2%, neighbor: 30.3% vs 11.4%) species both exhibited higher competitive frequencies compared to facilitation (Fig. S8a,d; Tables S15, S16). Furthermore, long‐lived species showed a higher frequency of competitive interactions than short‐lived species, both as focal species (95% CI [0.800, 1.133]) and as neighboring species (95% CI [0.866, 1.202]).

Co‐invasion reduced the performance of forb and grass species similarly, whereas woody species were not affected by co‐invasion (Fig. 4b,e; Tables S13, S14). However, neighboring woody species mitigated the negative effects of co‐invasion more strongly than forbs (Fig. 4e; Table S14). These patterns were mostly evident in growth metrics and were consistent for both focal and neighboring species (Figs 4b,e, S6d–f, S7d–f; Tables S13, S14). Life form also significantly influenced interaction types (*P* < 0.001; Fig. S8b,e; Tables S15, S16). Competitive interactions were more frequent than facilitation for forb (focal: 21.0% vs 9.5%, neighbor: 16.4% vs 12.7%) and grass (focal: 16.2% vs 13.0%, neighbor: 24.0% vs 8.1%), whereas woody species showed a higher frequency of facilitative interactions (focal: 14.5%; 95% CI [−0.973, 0.250]; neighbor: 16.3%; 95% CI [−0.562, 0.406]) (Fig. S8b,e; Tables S15, S16).

**Fig. 5:**
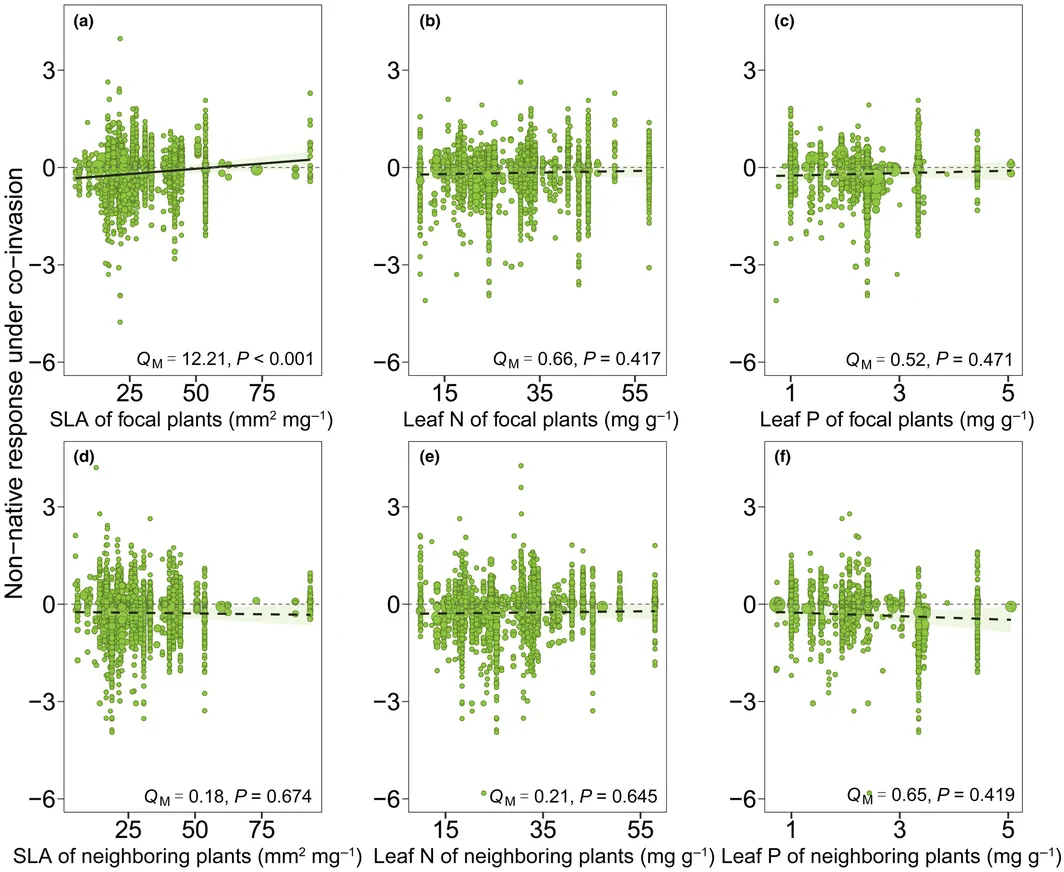
Effects of focal and neighboring non‐native plant functional traits on interaction outcomes under co‐invasion. (a) SLA, (b) leaf N content, and (c) leaf P content of focal non‐native plants. (d) SLA, (e) leaf N content, and (f) leaf P content of neighboring non‐native plants. The size of the points corresponds to the weight of each observation. The regression line indicates the response of the shaded prediction variable at the 95% confidence interval. The values of *Q*_M_ and *P* are displayed in each subplot. Solid lines represent significant relationships (*P* < 0.05), while dashed lines indicate non‐significant relationships (*P* > 0.05).

Most interactions (*c*. 75%) between nitrogen‐fixing and non‐nitrogen‐fixing species were neutral and mean competitive effects did not differ between focal nitrogen‐fixers and non‐fixers (Fig. 4c; Table S13). However, focal nitrogen‐fixing species had lower frequencies of both competition (11.4%; 95% CI [−0.999, −0.359]) and facilitation (8.5%; 95% CI [−0.868, −0.143]) than non‐fixers (competition: 19.1%, facilitation: 11.9%; Fig. S8c; Table S15). Non‐fixing neighbors exerted significant negative effects on focal species, whereas nitrogen‐fixing neighbors had neutral effects and were associated with higher focal performance (+11.2%; Fig. 4f; Table S14). Consistently, nitrogen‐fixing neighbors exerted higher facilitation (16.7%; 95% CI [0.064, 0.602]) and lower competition (9.3%; 95% CI [−1.187, −0.499]) frequency than non‐fixing neighbors (competition: 19.7%, facilitation: 11.1%) (Fig. S8f; Table S16). Results were qualitatively consistent in the full model (Table S17). However, among functional traits, only SLA significantly correlated with interactions among non‐native plants (Fig. 5), as the SLA of the focal non‐native plant increased, the net interaction became more positive (Fig. 5a).

**Fig. 6:**
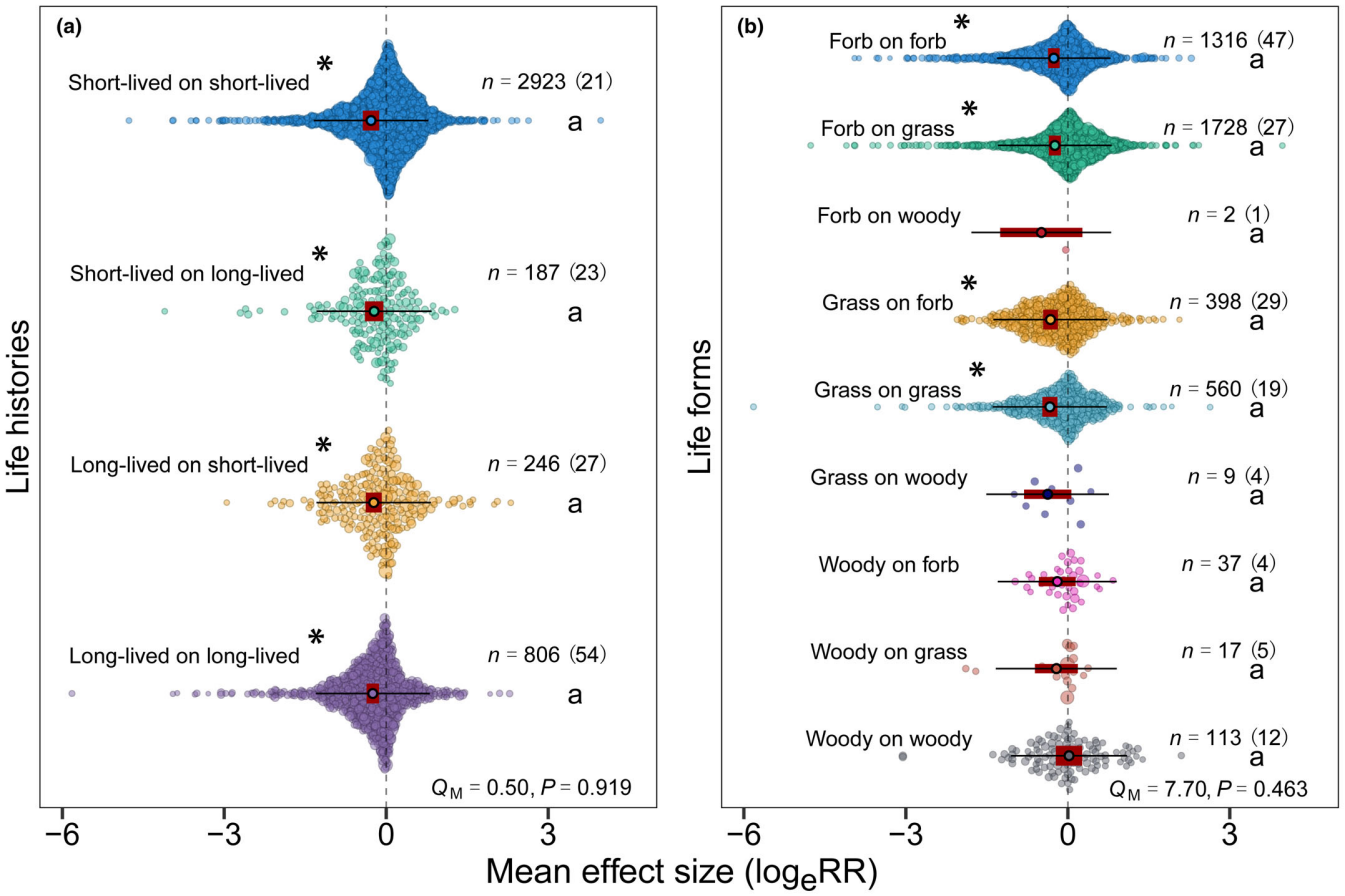
Interactive effects of trait combinations on non‐native plant performance under co‐invasion. Orchard plots show how combinations of (a) life‐history strategies and (b) life forms between focal and neighboring plants influence interaction outcomes. Points represent estimated means, while thick red whiskers indicate the 95% confidence intervals and thin black whiskers indicate 95% prediction intervals from multi‐level meta‐regression models. Asterisks denote significant effects. *n* represents the number of observations and experiments. The *Q*_M_ and *P*‐value are presented in each subplot. *P* < 0.05 indicates a significant difference between different factor levels and is marked with different letters.

Biological trait combinations between focal and neighboring plants showed consistently negative effects across life‐history pairings (Fig. 6a; Table S18). Forbs and grasses exerted significant negative effects on most life forms, whereas woody species had neutral effects on both similar and dissimilar life forms (Fig. 6b; Table S18).

### Non‐native plant richness, phylogeny and functional diversity as drivers of interactions among non‐native plants

**Fig. 7:**
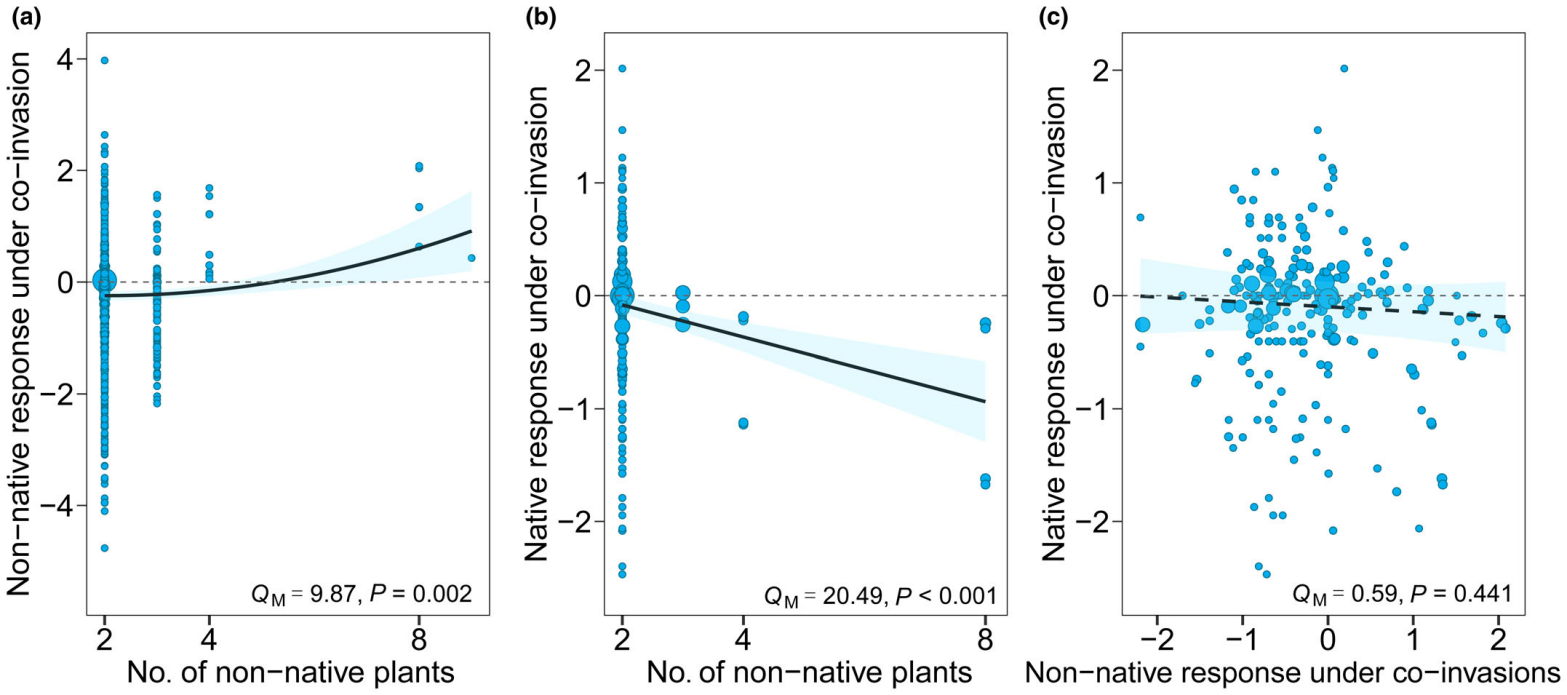
Effects of non‐native plant richness and native‐non‐native performance relationships on interaction outcomes under co‐invasion. (a) Effect of non‐native plant richness on non‐native plant performance under co‐invasion. (b) Negative effect of non‐native plant richness on native plant performance under co‐invasion. (c) Relationship between native plant performance and non‐native plant performance under co‐invasion. The size of the points corresponds to the weight of each observation. The regression line indicates the response of the shaded prediction variable at the 95% confidence interval. The values of *Q*_M_ and *P* are displayed in each subplot. Solid lines represent significant relationships (*P* < 0.05), while dashed lines indicate non‐significant relationships (*P* > 0.05).

Non‐native species richness affected interaction direction. When two or three non‐native species co‐occurred, performance was reduced relative to single‐species invasion (Table S19). However, as richness increased, negative effects weakened, shifting to neutral effects at a richness of four and to strong facilitation at a richness of eight or more (Figs 7a, S9; Table S19). Mean performance increased by 65.0% at a richness of four and 173.2% at a richness of eight or more, respectively, compared to the baseline of two species, indicating a clear non‐monotonic relationship (Fig. 7a). Replication at higher richness levels was limited. By contrast, native performance declined with increased non‐native richness (Fig. 7b). There was no significant relationship between native and non‐native responses under co‐invasion (Fig. 7c).

**Fig. 8:**
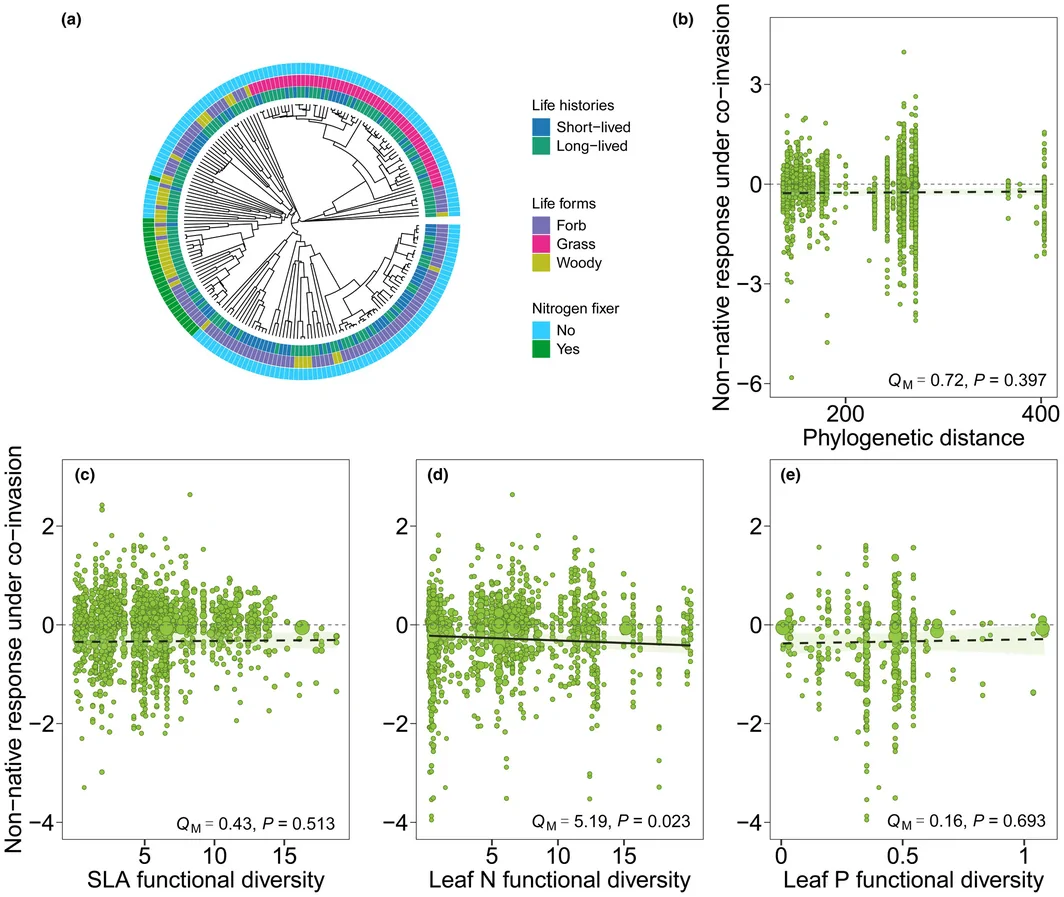
Effects of phylogenetic distance (PD) and functional diversity (FDis) on interaction outcomes under co‐invasion. (a) Phylogenetic tree of 202 non‐native plants included in the analysis. (b) Effect of phylogenetic distance on non‐native plant performance under co‐invasion. Effects of (c) SLA, (d) leaf N, and (e) leaf P functional diversity on non‐native plant performance under co‐invasion. The size of the points corresponds to the weight of each observation. The regression line indicates the response of the shaded prediction variable at the 95% confidence interval. The values of *Q*_M_ and *P* are displayed in each subplot. Solid lines represent significant relationships (*P* < 0.05), while dashed lines indicate non‐significant relationships (*P* > 0.05).

Phylogenetic analysis of the 202 non‐native species revealed no significant relationship between phylogenetic distance and interactions among co‐invaders (Fig. 8a,b). However, non‐native plant performance decreased significantly with increasing functional diversity (FDis) of leaf N content (Fig. 8d), whereas functional diversity based on SLA and leaf P content showed no significant effects (Fig. 8c,e).

### Assessment of publication bias

The funnel plot for the meta‐analysis was asymmetric (Fig. S10a), and Egger's regression indicated a significant relationship between effect size and sampling variance (log_e_RR = −0.335, 95% CI = [−0.431, −0.239]), suggesting potential publication bias among studies (Fig. S10b). However, Rosenberg's *N* was 955 589, well above the threshold of 22 085 (5*n* + 10); that is, the threshold for robust significant meta‐analytic results. We found no evidence of time‐lag bias, as effect sizes showed no significant relationship with publication year (log_e_RR = 0.008, 95% CI = [−0.004, 0.020]; Fig. S10c).

## Discussion

Field surveys indicated that multiple non‐native plant species frequently co‐occur within invaded communities, whereas the meta‐analysis suggested that most co‐invading plants have no effect on each other, or what we call neutral effects. Thus, the widespread occurrence of co‐invasion in natural communities cannot be interpreted as evidence for pervasive facilitation among invaders. The common neutral outcome was striking, as was the relatively low frequency of competitive outcomes, since the frequency of significant competitive outcomes among native species is generally several times higher (Keddy *et al*., 1998; Kuebbing & Nuñez, 2015; Maron *et al*., 2016; Adler *et al*., 2018; Golivets & Wallin, 2018). Also, in a meta‐analysis of interactions in general, Adler *et al*. (2018) found that interspecific competition was two to three times more common than facilitation, again demonstrating the relative lack of competition we found among non‐natives. However, by integrating a larger and more comprehensive dataset (4415 observations from 122 experiments) than previous meta‐analyses (Kuebbing & Nuñez, 2015, 2016; Golivets & Wallin, 2018), we found that the large number of neutral interactions and low number of negative interactions among non‐native invaders were also very different than interactions reported between invaders and native species. For example, Luo *et al*. (2025) found that seven of nine invasive species strongly suppressed natives in experiments, and Kuebbing & Nuñez (2016) reported that ‘non‐native plants were always bad neighbors’ for natives. Thus, in general, our findings indicate that interactions among co‐invaders yield much weaker and more inconsistent outcomes than interactions among invaders and native species.

### The paradox of prevalent co‐invasion under net competition

The overall mean intensity of interaction outcomes was competitive, and this is consistent with the literature (Kuebbing & Nuñez, 2015). However, facilitative interactions were consistently observed in certain contexts, suggesting that synergistic effects among non‐native plants should not be overlooked. Facilitation, or invasional meltdown, appeared to be especially relevant for woody and nitrogen‐fixing plants, likely driven by resource‐mediated mechanisms such as shading or nitrogen enrichment (Gómez‐Aparicio, 2009), which may promote the invasion of neighboring non‐native plants. In addition, facilitative interactions were more common in spatial associations, in certain ecosystems, and different life histories and life forms, likely due to niche differentiation and complementary resource utilization (He *et al*., 2013). Although facilitation was slightly less frequent overall than competition and neutral, their ecological consequences may be disproportionate to their occurrence. Therefore, facilitation among co‐invaders warrants particular attention. Non‐native plants are commonly better competitors than native plants (Vilà & Weiner, 2004; Kuebbing & Nuñez, 2016; Luo *et al*., 2025). When non‐native plants exerted stronger competitive pressure on native plants than on other non‐natives, the suppression of natives can indirectly facilitate the persistence of other invaders (Brooker *et al*., 2008; Kuebbing & Nuñez, 2016). Furthermore, an overall competitive mean outcome may explain why the removal of non‐native plants often triggers the competitive release of other non‐native plants (Emery *et al*., 2013; Ahmad *et al*., 2021).

We did not consider conditionality in the outcome of interactions among non‐natives in the context of resources or abiotic stress. Interactions among non‐native plant species tend to be more competitive at low abiotic stress than when stress is high (He *et al*., 2013). Likewise, as resource availability increases, non‐native plants may promote the growth of other non‐native plants (Sun *et al*., 2024). In addition, due to increased density, density‐dependent variation in resource availability may further intensify resource competition among non‐native plants, as we found that additive designs exhibit higher competition intensity compared to replacement designs. Facilitation among non‐native plants can have negative effects on natives, but we found no evidence for this. As the intensity of interactions among non‐natives shifted from strong competition to strong facilitation in co‐invasion, there was no significant and concomitant increase in the suppression of natives (Fig. 7c). This raises an important point in the study of co‐invasions. If co‐invaders demonstrate facilitation among each other without increasing impact, concern over co‐invasions, *per se*, may be less warranted. However, relatively few studies in our dataset explicitly quantified native responses to co‐invasion, limiting our ability to detect subtle effects in native suppression. Nevertheless, such effects may still emerge under particular environmental conditions or in specific ecological contexts. Future studies that simultaneously measure interactions among co‐invading non‐native species and their impacts on native species will be important for resolving when facilitation among invaders translates into greater ecological harm.

### Soil feedbacks modulate interaction outcomes

When plants compete directly with neighboring plants, they simultaneously engage in complex indirect interactions with the soil environment in ways that feedback to themselves and their neighbors (Lekberg *et al*., 2018). Here, indirect interactions among non‐natives mediated by plant–soil feedbacks produced a neutral mean, suggesting that, on average, indirect soil‐mediated effects among non‐natives are weak. However, plant–soil feedback slightly increased the frequency of competitive interactions and decreased the frequency of facilitative interactions, but did not translate into a stronger inhibitory effect on heterospecific non‐native species. This is consistent with literature showing that non‐native invaders commonly experience much weaker conspecific plant–soil feedback than native species or in soil from non‐native ranges (Callaway *et al*., 2004; Inderjit & van der Putten, 2010), and that soil biota can weaken competition among non‐natives (Zhang *et al*., 2020; Chen & van Kleunen, 2022). Therefore, soil‐mediated indirect interactions may modulate the frequency and reduce the intensity of interactions among non‐native species, thereby buffering their net effects and promoting coexistence.

### Traits shape interaction outcomes among co‐invading plants

The outcomes of co‐invasion corresponded with biological traits of the interacting species. Life histories and life forms are key mediators of plant–plant interactions in general (Gómez‐Aparicio, 2009; Kuebbing & Nuñez, 2015). Short‐lived annual species often exert stronger competitive effects than perennials due to their rapid resource utilization efficiency (Salguero‐Gómez *et al*., 2016; Shaukat *et al*., 2017). This general competitive advantage can be highly intensified at high resource levels (Besaw *et al*., 2011). Consistent with this, Kuebbing & Nuñez (2015) found that annual non‐native plant species were more frequently competitive than perennials. In our analysis, the life histories of non‐native plants did not change the intensity of mean negative interactions, with the exception that perennial non‐natives exerted weaker negative effects on germination. This suggests that the influence of life histories on interactions among co‐invading species may be most evident during early life stages, which are often particularly sensitive to biotic interactions (Schiffers & Tielbörger, 2006). In this context, most experiments in our meta‐analysis lasted less than 1 yr, thus, long‐term competitive outcomes may have been underestimated.

Herbaceous and graminoid co‐invaders consistently competed strongly, both in mean outcome of intensity and in frequency. By contrast, woody co‐invaders had no mean effect on each other, but focal woody species experienced more frequent competitive interactions, while neighboring woody species exerted more frequent facilitative interactions (Fig. S8b,e). These effects may be strongly affected by life stage, as woody perennials are often facilitated when young but switch to facilitators when mature (Callaway, 2007). In a multi‐biome study of *Prosopis juliflora*, a globally invasive tree, non‐native herbaceous plants in the understory were consistently facilitated while natives were consistently suppressed (Majumdar *et al*., 2025).

Non‐native species associated with nitrogen‐fixing mutualists diminished competition with other co‐invaders, perhaps through the facilitative effects of increased nitrogen supply, which can alleviate nutrient limitation and reduce direct resource competition among neighboring non‐native plants (Carino & Daehler, 2002; Ren *et al*., 2023). By enhancing the availability of soil nitrogen (nitrogen‐fixing species) or creating other favorable micro‐environments (such as the shade provided by woody species) (Gómez‐Aparicio, 2009), the interactions between co‐invading species may shift from competitive to neutral or facilitative outcomes. Similarly, we found that the performance of focal non‐native plants increased with higher SLA, indicating that acquisitive resource‐use strategies can confer a competitive advantage under co‐invasion. Species with high SLA are typically associated with rapid resource acquisition, high photosynthetic rates, and fast growth, which may allow them to pre‐empt light and nutrient resources more effectively than their neighbors (Díaz *et al*., 2016; Feng & van Kleunen, 2016). In this case, competitive outcomes may be driven more by differences in resource acquisition efficiency than by niche differentiation *per se*.

### Richness and phylogenetic and functional drivers of interactions among non‐native species

We found that as non‐native plant richness increased, interactions among non‐native species shifted from competitive to facilitative. This suggests that non‐native species richness may likewise promote invasions through mechanisms analogous to complementarity and selection effects (Wilsey *et al*., 2009; Isbell & Wilsey, 2011; Kuebbing *et al*., 2015b). In some cases, greater non‐native richness can reduce interspecific competition and increase facilitation among non‐native species and could intensify competition with native species (Wang *et al*., 2022). But these changes in interactions among co‐invaders were not accompanied by stronger suppression of native species across richness levels overall. However, native species experienced intensifying competition as non‐native species richness increased, which is consistent with previous studies (Kaul & Wilsey, 2021; Wang *et al*., 2022). These richness effects in interaction outcomes may partly contribute to the widespread occurrence of multispecies co‐invasion observed in natural communities.

Consistent with previous studies (Soliveres *et al*., 2014; Ferenc & Sheppard, 2020), our results show that the direction and intensity of interactions among non‐native plants are not correlated with phylogenetic distance. Phylogenetic distance is an integrated measure; therefore, it may not perfectly reflect stabilizing niche differences and average fitness differences between species (Narwani *et al*., 2013; Godoy *et al*., 2014). On the one hand, greater evolutionary divergence may be associated with stronger stabilizing niche differences, for example, resource allocation or host‐specific predators, which can reduce intraspecific competition and promote coexistence (HilleRisLambers *et al*., 2012). On the other hand, it may also reflect greater differences in competitive ability (average fitness differences), which would exacerbate competitive asymmetry and lead to competitive exclusion (Mayfield & Levine, 2010). Due to these opposing mechanisms, their combined effects may mask any consistent relationship between phylogenetic distance and interactions. By contrast, functional traits may provide a more direct link to the mechanisms underlying species interactions. Traits such as SLA and leaf N content are closely related to resource utilization strategies and thus may better capture stabilizing niche differences and average fitness differences among co‐invading species (Díaz *et al*., 2016; Feng & van Kleunen, 2016). We found that the performance of non‐native plants declined with increasing leaf N functional diversity, suggesting that greater variation in nutrient utilization strategies may exacerbate competitive asymmetry among co‐invading species. Communities with high leaf N diversity may contain species with different nutrient utilization strategies, which could lead to unequal competitive advantages and more strongly suppress less competitive species (Mayfield & Levine, 2010). Furthermore, higher leaf N variability may reflect increased heterogeneity in resource demand, which could exacerbate competition under nutrient‐limited conditions rather than promote complementarity (Kunstler *et al*., 2012).

### Limitations and future perspectives

There are several limitations and caveats that warrant consideration. First, although we analyzed the effects of a wide range of experimental conditions, functional traits, and phylogenetic relationships, substantial variation remains unexplained, suggesting the potential importance of unmeasured factors such as fine‐scale environmental gradients, additional functional traits, herbivory pressure, or soil physicochemical properties. Second, the dataset is unequally distributed across response types and richness of non‐native plants. In particular, few studies have examined whether the germination and reproduction of non‐native plants are directly or indirectly influenced by other non‐native competitors. In addition, most of the included experiments focused on interactions between two or three non‐native plants, whereas far fewer studies have addressed co‐invasion in more diverse communities or associated changes in co‐occurring native plants. Therefore, future research should examine how germination and reproduction of non‐native plants respond to multiple non‐native competitors within more diverse communities. Third, the predominance of short‐term experiments (typically < 1 yr) in our dataset constrains inference to initial interaction phases, whereas in natural systems, invaders may persist for decades. In ecosystems lacking management or intervention, non‐native species often establish long‐term persistence, gradually forming more complex, stable, and even feedback‐enhanced interactions with other plants. Therefore, long‐term experiments are needed to determine whether the outcomes of interactions among non‐native plants will persist, intensify, or reverse over time.

### Implications for predicting and managing invasion complexes

Since the introduction of the invasional meltdown hypothesis by Simberloff & Von Holle (1999), more attention has been paid to interactions across trophic levels (Jackson, 2015; Braga *et al*., 2018) than to facilitation among non‐native plants. Our field surveys showed that co‐occurrence among non‐native plants is widespread in invaded communities, whereas our meta‐analysis revealed that interactions among co‐invading plant species are predominantly neutral rather than strongly facilitative. Competition and facilitation occurred less frequently and were highly context‐dependent. Increasing non‐native richness shifted interactions from competition to facilitation, while also intensifying negative effects on co‐occurring native species. By contrast, phylogenetic distance did not predict interaction outcomes, whereas functional diversity, particularly in SLA and leaf N, significantly influenced non‐native performance, indicating that functional traits better capture interaction mechanisms. Together, these findings suggest that invasional meltdown is not a universal outcome of co‐invasion by plants, but depends on species composition, functional traits, and community context.

## Competing interests

None declared.

## Author contributions

XS and FH conceived and designed the research. ZY, XT and YC conducted field surveys and compiled field‐based datasets included in the synthesis. FH, YS and XZ conducted literature screening and data extraction and curated the meta‐analytic database. XS, RMC and FH developed the conceptual structure. FH performed the analysis and wrote the first draft of the manuscript. XS and RMC contributed to the interpretation of the results and manuscript revision. All authors participated in revisions and approved the final version of the manuscript.

## Disclaimer

The New Phytologist Foundation remains neutral with regard to jurisdictional claims in maps and in any institutional affiliations.

## Supporting information


**Fig. S1** PRISMA flow diagram for meta‐analysis.
**Fig. S2** Effects of experimental context and ecosystem type on the interaction proportions of non‐native plants.
**Fig. S3** Effects of plant removal experiments on non‐native plant performance and the interaction proportions under co‐invasion.
**Fig. S4** Different responses of non‐native plants to co‐invasion by ecosystem.
**Fig. S5** Relationship between the performance of non‐native plants under co‐invasion and experimental duration.
**Fig. S6** Different responses of non‐native plants to co‐invasion by biological traits of focal plants.
**Fig. S7** Different responses of non‐native plants to co‐invasion by biological traits of neighboring plants.
**Fig. S8** Effects of biological traits of focal and neighboring plants on the interaction proportions of non‐native plants.
**Fig. S9** Effects of non‐native plant richness on performance under co‐invasion.
**Fig. S10** Assessment of publication bias in the meta‐analysis.
**Methods S1** Definition and classification of interactions among non‐native plants in analyzed studies.
**Table S1** Distribution of observations and experiments by continent.
**Table S2** Distribution of observations and experiments by ecosystem type.
**Table S3** Distribution of observations and experiments by experiment type.
**Table S4** Distribution of observations and experiments by experiment design.
**Table S5** Distribution of observations and experiments by non‐native plant richness studies.
**Table S6** Distribution of observations and experiments by removal experiment status.
**Table S7** Distribution of observations and experiments by feedback experiment status.
**Table S8** The number of observations on non‐native plant performance under co‐invasion across all experiments.
**Table S9** Distribution of interaction direction among co‐invading non‐native species.
**Table S10** Effects of moderator variables on the performance of non‐native plants under co‐invasion.
**Table S11** The proportion of interaction types among non‐native plants in different experimental contexts and ecosystems.
**Table S12** Results of a full mixed‐effects meta‐analysis model simultaneously testing multiple moderator variables (experimental contexts) affecting non‐native plant performance.
**Table S13** Effects of focal plant biological traits on non‐native plant performance under co‐invasion.
**Table S14** Effects of neighboring plant biological traits on non‐native plant performance under co‐invasion.
**Table S15** The proportion of interaction types among focal non‐native plants in different biological traits.
**Table S16** The proportion of interaction types among neighboring non‐native plants in different biological traits.
**Table S17** Results of a full mixed‐effects meta‐analysis model simultaneously testing multiple moderator variables (biological traits) affecting non‐native plant performance.
**Table S18** Effects of trait combinations of focal and neighboring plant on non‐native plant performance under co‐invasion.
**Table S19** Effects of non‐native plant richness on alien plant performance under co‐invasion.
Please note: Wiley is not responsible for the content or functionality of any Supporting Information supplied by the authors. Any queries (other than missing material) should be directed to the *New Phytologist* Central Office.

## Data Availability

Data and R code associated with this study are publicly accessible in figshare (doi: 10.6084/m9.figshare.32778642).

## References

[nph71463-bib-0001] Adler PB , Smull D , Beard KH , Choi RT , Furniss T , Kulmatiski A , Meiners JM , Tredennick AT , Veblen KE . 2018. Competition and coexistence in plant communities: intraspecific competition is stronger than interspecific competition. Ecology Letters 21: 1319–1329. 29938882 10.1111/ele.13098

[nph71463-bib-0002] Ahmad R , Lone SA , Rashid I , Khuroo AA . 2025. A global synthesis of the ecological effects of co‐invasions. Journal of Ecology 113: 570–581.

[nph71463-bib-0003] Ahmad R , Rashid I , Hamid M , Malik AH , Khuroo AA . 2021. Invasion shadows in soil system overshadow the restoration of invaded ecosystems: implications for invasive plant management. Ecological Engineering 164: 106219.

[nph71463-bib-0004] Bertness MD , Callaway RM . 1994. Positive interactions in communities. Trends in Ecology & Evolution 9: 191–193. 21236818 10.1016/0169-5347(94)90088-4

[nph71463-bib-0005] Besaw LM , Thelen GC , Sutherland S , Metlen K , Callaway RM . 2011. Disturbance, resource pulses and invasion: short‐term shifts in competitive effects, not growth responses, favour exotic annuals. Journal of Applied Ecology 48: 998–1006.

[nph71463-bib-0006] Bonanomi G , Incerti G , Mazzoleni S . 2011. Assessing occurrence, specificity, and mechanisms of plant facilitation in terrestrial ecosystems. Plant Ecology 212: 1777–1790.

[nph71463-bib-0007] Borenstein M , Hedges LV , Higgins JPT , Rothstein HR . 2009. Introduction to meta‐analysis. West Sussex, UK: Wiley, New York.

[nph71463-bib-0008] Braga RR , Gómez‐Aparicio L , Heger T , Simões Vitule JR , Jeschke JM . 2018. Structuring evidence for invasional meltdown: broad support but with biases and gaps. Biological Invasions 20: 923–936.

[nph71463-bib-0009] Brandt AJ , Png GK , Jo I , McGrannachan C , Allen K , Peltzer DA , D'Antonio C , Dickie IA , French K , Leishman MR *et al*. 2023. Managing multi‐species plant invasions when interactions influence their impact. Frontiers in Ecology and the Environment 21: 370–379.

[nph71463-bib-0010] Brooker RW , Maestre FT , Callaway RM , Lortie CL , Cavieres LA , Kunstler G , Liancourt P , Tielboerger K , Travis JMJ , Anthelme F *et al*. 2008. Facilitation in plant communities: the past, the present, and the future. Journal of Ecology 96: 18–34.

[nph71463-bib-0011] Bürkner P‐C . 2017. brms: an R package for Bayesian multilevel models using Stan. Journal of Statistical Software 80: 1–28.

[nph71463-bib-0012] Cadotte MW , Campbell SE , Li S‐P , Sodhi DS , Mandrak NE . 2018. Preadaptation and naturalization of nonnative species: Darwin's two fundamental insights into species invasion. Annual Review of Plant Biology 69: 661–684. 10.1146/annurev-arplant-042817-040339 29489400

[nph71463-bib-0013] Callaway RM . 2007. Positive interactions and interdependence in plant communities. Dordrecht, The Netherlands: Springer Science & Business Media.

[nph71463-bib-0014] Callaway RM , Thelen GC , Rodriguez A , Holben WE . 2004. Soil biota and exotic plant invasion. Nature 427: 731–733. 14973484 10.1038/nature02322

[nph71463-bib-0015] Callaway RM , Walker LR . 1997. Competition and facilitation: a synthetic approach to interactions in plant communities. Ecology 78: 1958–1965.

[nph71463-bib-0016] Carino DA , Daehler CC . 2002. Can inconspicuous legumes facilitate alien grass invasions? Partridge peas and fountain grass in Hawai’i. Ecography 25: 33–41.

[nph71463-bib-0017] Chamberlain S , Szoecs E , Foster Z , Arendsee Z , Boettiger C , Ram K , Bartomeus I , Baumgartner J , O'Donnell J , Oksanen J . 2020. taxize: taxonomic information from around the web . R package v.0.9 92. https://docs.ropensci.org/taxize/.

[nph71463-bib-0018] Chen C , Xiao W , Chen HYH . 2025. Meta‐analysis reveals global variations in plant diversity effects on productivity. Nature 638: 435–440. 39779865 10.1038/s41586-024-08407-8

[nph71463-bib-0019] Chen D , van Kleunen M . 2022. Invasional meltdown mediated by plant–soil feedbacks may depend on community diversity. New Phytologist 235: 1589–1598. 35551668 10.1111/nph.18218

[nph71463-bib-0020] Darwin C . 1859. On the origin of species. London, UK: John Murray.

[nph71463-bib-0021] Díaz S , Kattge J , Cornelissen JHC , Wright IJ , Lavorel S , Dray S , Reu B , Kleyer M , Wirth C , Prentice IC *et al*. 2016. The global spectrum of plant form and function. Nature 529: 167–171. 26700811 10.1038/nature16489

[nph71463-bib-0022] Emery SM , Doran PJ , Legge JT , Kleitch M , Howard S . 2013. Aboveground and belowground impacts following removal of the invasive species baby's breath (*Gypsophila paniculata*) on Lake Michigan sand dunes. Restoration Ecology 21: 506–514.

[nph71463-bib-0023] Faith DP . 1992. Conservation evaluation and phylogenetic diversity. Biological Conservation 61: 1–10.

[nph71463-bib-0024] Feng YH , van Kleunen M . 2016. Phylogenetic and functional mechanisms of direct and indirect interactions among alien and native plants. Journal of Ecology 104: 1136–1148.

[nph71463-bib-0025] Ferenc V , Sheppard CS . 2020. The stronger, the better – trait hierarchy is driving alien species interaction. Oikos 129: 1455–1467.

[nph71463-bib-0026] Flory SL , Bauer JT . 2014. Experimental evidence for indirect facilitation among invasive plants. Journal of Ecology 102: 12–18.

[nph71463-bib-0027] Godoy O , Kraft NJB , Levine JM . 2014. Phylogenetic relatedness and the determinants of competitive outcomes. Ecology Letters 17: 836–844. 24766326 10.1111/ele.12289

[nph71463-bib-0028] Golivets M , Wallin KF . 2018. Neighbour tolerance, not suppression, provides competitive advantage to non‐native plants. Ecology Letters 21: 745–759. 29516604 10.1111/ele.12934

[nph71463-bib-0029] Gómez‐Aparicio L . 2009. The role of plant interactions in the restoration of degraded ecosystems: a meta‐analysis across life‐forms and ecosystems. Journal of Ecology 97: 1202–1214.

[nph71463-bib-0030] He Q , Bertness MD , Altieri AH . 2013. Global shifts towards positive species interactions with increasing environmental stress. Ecology Letters 16: 695–706. 23363430 10.1111/ele.12080

[nph71463-bib-0031] HilleRisLambers J , Adler PB , Harpole WS , Levine JM , Mayfield MM . 2012. Rethinking community assembly through the lens of coexistence theory. Annual Review of Ecology, Evolution, and Systematics 43: 227–248.

[nph71463-bib-0032] Inderjit , van der Putten WH . 2010. Impacts of soil microbial communities on exotic plant invasions. Trends in Ecology & Evolution 25: 512–519. 20638747 10.1016/j.tree.2010.06.006

[nph71463-bib-0033] Isbell FI , Wilsey BJ . 2011. Increasing native, but not exotic, biodiversity increases aboveground productivity in ungrazed and intensely grazed grasslands. Oecologia 165: 771–781. 21161547 10.1007/s00442-010-1877-9

[nph71463-bib-0034] Jackson MC . 2015. Interactions among multiple invasive animals. Ecology 96: 2035–2041. 26405728 10.1890/15-0171.1

[nph71463-bib-0035] Jin Y , Qian H . 2022. v.phylomaker2: an updated and enlarged R package that can generate very large phylogenies for vascular plants. Plant Diversity 44: 335–339. 35967255 10.1016/j.pld.2022.05.005 PMC9363651

[nph71463-bib-0036] Kattge J , Bönisch G , Díaz S , Lavorel S , Prentice IC , Leadley P , Tautenhahn S , Werner GDA , Aakala T , Abedi M *et al*. 2020. TRY plant trait database ‐ enhanced coverage and open access. Global Change Biology 26: 119–188. 31891233 10.1111/gcb.14904

[nph71463-bib-0037] Kaul AD , Wilsey BJ . 2021. Exotic species drive patterns of plant species diversity in 93 restored tallgrass prairies. Ecological Applications 31: e2252. 33145856 10.1002/eap.2252

[nph71463-bib-0038] Keddy P , Fraser LH , Wisheu IC . 1998. A comparative approach to examine competitive response of 48 wetland plant species. Journal of Vegetation Science 9: 777–786.

[nph71463-bib-0039] van Kleunen M , Dawson W , Essl F , Pergl J , Winter M , Weber E , Kreft H , Weigelt P , Kartesz J , Nishino M *et al*. 2015. Global exchange and accumulation of non‐native plants. Nature 525: 100–103. 26287466 10.1038/nature14910

[nph71463-bib-0040] van Kleunen M , Pysek P , Dawson W , Essl F , Kreft H , Pergl J , Weigelt P , Stein A , Dullinger S , König C *et al*. 2019. The Global Naturalized Alien Flora (GloNAF) database. Ecology 100: e02542. 41195458 10.1002/ecy.70245

[nph71463-bib-0041] Koricheva J , Gurevitch J , Mengersen K . 2013. Handbook of meta‐analysis in ecology and evolution. Princeton, NJ, USA: Princeton University Press.

[nph71463-bib-0042] Kuebbing SE , Classen AT , Call JJ , Henning JA , Simberloff D . 2015a. Plant–soil interactions promote co‐occurrence of three nonnative woody shrubs. Ecology 96: 2289–2299. 26405753 10.1890/14-2006.1

[nph71463-bib-0043] Kuebbing SE , Classen AT , Sanders NJ , Simberloff D . 2015b. Above‐ and below‐ground effects of plant diversity depend on species origin: an experimental test with multiple invaders. New Phytologist 208: 727–735. 26053089 10.1111/nph.13488

[nph71463-bib-0044] Kuebbing SE , Nuñez MA . 2015. Negative, neutral, and positive interactions among nonnative plants: patterns, processes, and management implications. Global Change Biology 21: 926–934. 25142018 10.1111/gcb.12711

[nph71463-bib-0045] Kuebbing SE , Nuñez MA . 2016. Invasive non‐native plants have a greater effect on neighbouring natives than other non‐natives. Nature Plants 2: 1–7. 10.1038/nplants.2016.134 27618506

[nph71463-bib-0046] Kuebbing SE , Nuñez MA , Simberloff D . 2013. Current mismatch between research and conservation efforts: the need to study co‐occurring invasive plant species. Biological Conservation 160: 121–129.

[nph71463-bib-0047] Kunstler G , Lavergne S , Courbaud B , Thuiller W , Vieilledent G , Zimmermann NE , Kattge J , Coomes DA . 2012. Competitive interactions between forest trees are driven by species' trait hierarchy, not phylogenetic or functional similarity: implications for forest community assembly. Ecology Letters 15: 831–840. 22625657 10.1111/j.1461-0248.2012.01803.x PMC4003531

[nph71463-bib-0048] Laliberté E , Legendre P . 2010. A distance‐based framework for measuring functional diversity from multiple traits. Ecology 91: 299–305. 20380219 10.1890/08-2244.1

[nph71463-bib-0049] Lekberg Y , Bever JD , Bunn RA , Callaway RM , Hart MM , Kivlin SN , Klironomos J , Larkin BG , Maron JL , Reinhart KO *et al*. 2018. Relative importance of competition and plant‐soil feedback, their synergy, context dependency and implications for coexistence. Ecology Letters 21: 1268–1281. 29896848 10.1111/ele.13093

[nph71463-bib-0050] Luo WB , Liao HX , Callaway R , Pal RW . 2025. Competition on a neutral playing field: invaders still win and size still matters… sometimes. Proceedings of the Royal Society B‐Biological Sciences 292: 20250087. 10.1098/rspb.2025.0087 PMC11936679 40132634

[nph71463-bib-0051] Ma J , Li H . 2018. The checklist of the alien invasive plants in China. Beijing, China: Higher Education Press.

[nph71463-bib-0052] Majumdar S , Kaur S , Liao H , Kaur L , Pysek P , Pergl J , Cadotte MW , Callaway RM , Inderjit . 2025. Invasive *Prosopis* associated with large, but idiosyncratic, ecosystem changes and loss of native biodiversity across India. Plant Ecology 226: 1315–1333.

[nph71463-bib-0053] Maron JL , Smith AL , Ortega YK , Pearson DE , Callaway RM . 2016. Negative plant‐soil feedbacks increase with plant abundance, and are unchanged by competition. Ecology 97: 2055–2063. 27859206 10.1002/ecy.1431

[nph71463-bib-0054] Martorell C , Martinez‐Blancas A , Garcia‐Meza D . 2021. Plant‐soil feedbacks depend on drought stress, functional group, and evolutionary relatedness in a semiarid grassland. Ecology 102: e03499. 34314034 10.1002/ecy.3499

[nph71463-bib-0055] Mayfield MM , Levine JM . 2010. Opposing effects of competitive exclusion on the phylogenetic structure of communities. Ecology Letters 13: 1085–1093. 20576030 10.1111/j.1461-0248.2010.01509.x

[nph71463-bib-0056] Myster RW , Pickett STA . 1992. Dynamics of association between plants in ten old fields during 31 years of succession. Journal of Ecology and Environment 80: 291–302.

[nph71463-bib-0057] Nakagawa S , Lagisz M , O'Dea RE , Pottier P , Rutkowska J , Senior AM , Yang Y , Noble DWA . 2023. orchard 2.0: an R package for visualising meta‐analyses with orchard plots. Methods in Ecology and Evolution 14: 2003–2010.

[nph71463-bib-0058] Narwani A , Alexandrou MA , Oakley TH , Carroll IT , Cardinale BJ . 2013. Experimental evidence that evolutionary relatedness does not affect the ecological mechanisms of coexistence in freshwater green algae. Ecology Letters 16: 1373–1381. 24112458 10.1111/ele.12182

[nph71463-bib-0059] O'Dea RE , Lagisz M , Jennions MD , Koricheva J , Noble DWA , Parker TH , Gurevitch J , Page MJ , Stewart G , Moher D *et al*. 2021. Preferred reporting items for systematic reviews and meta‐analyses in ecology and evolutionary biology: a PRISMA extension. Biological Reviews 96: 1695–1722. 33960637 10.1111/brv.12721 PMC8518748

[nph71463-bib-0060] Pyšek P , Hulme PE , Simberloff D , Bacher S , Blackburn TM , Carlton JT , Dawson W , Essl F , Foxcroft LC , Genovesi P *et al*. 2020. Scientists' warning on invasive alien species. Biological Reviews 95: 1511–1534. 32588508 10.1111/brv.12627 PMC7687187

[nph71463-bib-0061] Pyšek P , Jarošík V , Hulme PE , Pergl J , Hejda M , Schaffner U , Vilà M . 2012. A global assessment of invasive plant impacts on resident species, communities and ecosystems: the interaction of impact measures, invading species' traits and environment. Global Change Biology 18: 1725–1737.

[nph71463-bib-0062] Rauschert ESJ , Shea K . 2012. Invasional interference due to similar inter‐ and intraspecific competition between invaders may affect management. Ecological Applications 22: 1413–1420. 22908701 10.1890/11-2107.1

[nph71463-bib-0063] Reinhart KO , Callaway RM . 2006. Soil biota and invasive plants. New Phytologist 170: 445–457. 16626467 10.1111/j.1469-8137.2006.01715.x

[nph71463-bib-0064] Ren J , Chen P , Shen C , Tao Z , Huang W . 2023. Functional and phylogenetic similarities of co‐occurring invaders affect the growth of an invasive forb. Journal of Plant Ecology 16: rtad007.

[nph71463-bib-0065] Salguero‐Gómez R , Jones OR , Jongejans E , Blomberg SP , Hodgson DJ , Mbeau‐Ache C , Zuidema PA , de Kroon H , Buckley YM . 2016. Fast‐slow continuum and reproductive strategies structure plant life‐history variation worldwide. Proceedings of the National Academy of Sciences, USA 113: 230–235. 10.1073/pnas.1506215112 PMC4711876 26699477

[nph71463-bib-0066] Schiffers K , Tielbörger K . 2006. Ontogenetic shifts in interactions among annual plants. Journal of Ecology 94: 336–341.

[nph71463-bib-0067] Seebens H , Bacher S , Blackburn TM , Capinha C , Dawson W , Dullinger S , Genovesi P , Hulme PE , van Kleunen M , Kuhn I *et al*. 2020. Projecting the continental accumulation of alien species through to 2050. Global Change Biology 27: 970–982. 10.1111/gcb.15333 33000893

[nph71463-bib-0068] Shaukat SS , Khan MA , Zafar H , Noor N , Alamgir A . 2017. Competitive interactions between a perennial legume shrub *Indigofera oblongifolia* and two annuals *Tephrosia strigosa* and *Corchorus trilocularis* . Pakistan Journal of Botany 49: 2383–2394.

[nph71463-bib-0069] Sheppard CS , Carboni M , Essl F , Seebens H , Thuiller W , DivGrass C . 2018. It takes one to know one: similarity to resident alien species increases establishment success of new invaders. Diversity and Distributions 24: 680–691.

[nph71463-bib-0070] Simberloff D , Von Holle B . 1999. Positive interactions of nonindigenous species: invasional meltdown? Biological Invasions 1: 21–32.

[nph71463-bib-0071] Soliveres S , Maestre FT , Bowker MA , Torices R , Quero JL , García‐Gómez M , Cabrera O , Cea AP , Coaguila D , Eldridge DJ *et al*. 2014. Functional traits determine plant co‐occurrence more than environment or evolutionary relatedness in global drylands. Perspectives in Plant Ecology, Evolution and Systematics 16: 164–173. 25914604 10.1016/j.ppees.2014.05.001 PMC4407970

[nph71463-bib-0072] Sun Y , Ren Z‐K , Muller‐Scharer H , Callaway RM , van Kleunen M , Huang W . 2024. Increasing and fluctuating resource availability enhances invasional meltdown. Ecology 105: e4387. 39016245 10.1002/ecy.4387

[nph71463-bib-0073] Thakur MP , Gu Z , van Kleunen M , Zhou X . 2025. Invasion impacts in terrestrial ecosystems: global patterns and predictors. Science 390: 381–385. 41129653 10.1126/science.adq3101

[nph71463-bib-0074] Thouvenot L , Puech C , Martinez L , Haury J , Thiebaut G . 2013. Strategies of the invasive macrophyte *Ludwigia grandiflora* in its introduced range: competition, facilitation or coexistence with native and exotic species? Aquatic Botany 107: 8–16.

[nph71463-bib-0075] Trikalinos TA , Ioannidis JP . 2005. Assessing the evolution of effect sizes over time. In: Rothstein HR , Sutton AJ , Borenstein M , eds. Publication Bias in Meta‐analysis: Prevention, Assessment and Adjustments. Chichester, UK: John Wiley & Sons Ltd, 241–260.

[nph71463-bib-0076] Viechtbauer W . 2010. Conducting meta‐analyses in R with the metafor package. Journal of Statistical Software 36: 1–48.

[nph71463-bib-0077] Viechtbauer W , Cheung MWL . 2010. Outlier and influence diagnostics for meta‐analysis. Research Synthesis Methods 1: 112–125. 26061377 10.1002/jrsm.11

[nph71463-bib-0078] Vilà M , Weiner J . 2004. Are invasive plant species better competitors than native plant species? ‐ Evidence from pair‐wise experiments. Oikos 105: 229–238.

[nph71463-bib-0079] Wagg C , Ebeling A , Roscher C , Ravenek J , Bachmann D , Eisenhauer N , Mommer L , Buchmann N , Hillebrand H , Schmid B *et al*. 2017. Functional trait dissimilarity drives both species complementarity and competitive disparity. Functional Ecology 31: 2320–2329.

[nph71463-bib-0080] Wang C‐Y , Li Y , Li C , Zhong S‐S , Xu Z‐L , Yu Y‐L , Du D‐L . 2023. A method for quantifying relative competitive advantage and the combined effect of co‐invasion for two invasive plants. Plant Diversity 45: 358–361. 37397593 10.1016/j.pld.2023.01.005 PMC10311150

[nph71463-bib-0081] Wang X , Wang J , Hu B , Zheng W‐L , Li M , Shen Z‐X , Yu F‐H , Schmid B , Li M‐H . 2022. Richness, not evenness, of invasive plant species promotes invasion success into native plant communities via selection effects. Oikos 2022: e08966.

[nph71463-bib-0082] Wilsey BJ , Teaschner TB , Daneshgar PP , Isbell FI , Polley HW . 2009. Biodiversity maintenance mechanisms differ between native and novel exotic‐dominated communities. Ecology Letters 12: 432–442. 19379137 10.1111/j.1461-0248.2009.01298.x

[nph71463-bib-0083] Zhang Z , Liu Y , Brunel C , van Kleunen M . 2020. Soil‐microorganism‐mediated invasional meltdown in plants. Nature Ecology & Evolution 4: 1612–1621. 33020599 10.1038/s41559-020-01311-0

