# Supplementary material for: Neutral interactions among non‐native plants are common
Source: New Phytol. 2026 Jul 24;252(1):292–309. doi: 10.1111/nph.71463 (PMC13539978; doi:10.1111/nph.71463)
Supplement: Supplementary file 1 [file NPH-252-292-s001.pdf]

***New Phytologist* Supporting Information**

Article title: Neutral interactions among non-native plants are common

Authors: Feng He, Zhiliang Yuan, Yumei Sun, Xincong Zhai, Xiangyu Tian, Yun Chen, Ragan M. Callaway, Xiao Sun

Article acceptance date: 8 July 2026

The following Supporting Information is available for this article:

Methods S1

Fig. S1-10

Table S1-19

List of studies included in our database

Methods S1 Definition and classification of interactions among non-native plants in analyzed studies.

(1) Direct interactions are those between two heterospecific or conspecific individuals, and not affected by other individuals or other species (Jones & Callaway, 2007).
(2) Addition experiments: Addition series studies compare the performance of focal plants in the absence of neighboring non-native plants to their performance in the presence of heterospecific non-native plants.
(3) Replacement experiments: Replacement series assess how the performance of a focal non-native species responds to changes in the conspecifics versus heterospecifics at a fixed total density, providing a measure of intra- and interspecific interactions.
(4) Removal experiments: Studies controlling interactions by removing neighboring plants. Removal experiments that manipulate the performance of focal plants by removing heterospecific plants and comparing them to controls without neighbors removed.
(5) Field surveys: Studies conducted at sites where neighboring non-native plants naturally either occur or are absent around the focal non-native plant.
(6) Plant richness experiments: Often referred to as “biodiversity-ecosystem function” experiments, these studies manipulate the species richness of plants and record community performance. These designs are considered to be direct interactions but usually do not control for the indirect effects of herbivores or soil biota. To more comprehensively incorporate experimental studies centered on non-native plant diversity gradients, we conducted supplementary searches beyond the initial literature review, focusing specifically on experimental research that explicitly manipulated or compared non-native plant species richness, diversity, or mixture composition. We conducted the search using the following keyword combinations: ("invasive plant* richness" OR "invasive plant* diversity" OR "invasive plant* mix*" OR "alien plant* richness" OR "alien plant* diversity" OR "alien plant* mix*" OR "exotic plant* richness" OR "exotic plant* diversity" OR "exotic plant* mix*" OR "non-native plant* richness" OR "non-native plant* diversity" OR "non-native plant* mix*" OR "inva* species* richness" OR "inva* species* diversity" OR "invasive species* mix*" OR "alien species* richness" OR "alien species* diversity" OR "alien species* mix*" OR "exotic species* richness" OR "exotic species* diversity" OR "exotic species* mix*" OR "non-native species* richness" OR "non-native species* diversity" OR "non-native species* mix*" OR "inva* richness" OR "inva* diversity" OR "invasive mix*" OR "alien richness" OR "alien diversity" OR "alien mix*" OR "exotic richness" OR "exotic

diversity" OR "exotic mix*" OR "non-native richness" OR "non-native diversity" OR "non-native mix*") AND (experiment* OR manipulat* OR assembl* OR gradient OR treatment). Through this supplementary search, we systematically identified and incorporated experimental studies centered on non-native plant diversity gradients, thereby ensuring this research type is fully represented in the dataset.

Indirect interactions were restricted to plant-soil feedback studies, in which one non-native plant altered soil biotic or abiotic properties that subsequently affected the performance of another non-native plant, without any direct physical contact. Although indirect interactions in ecological theory can broadly include effects mediated by third-party species or processes (Strauss, 1991), our synthesis focuses exclusively on soil-mediated pathways that can be explicitly quantified across studies.

In our meta-analysis, some studies included both native and non-native plants. We only included such studies when the design allowed the interactions among non-native species to be clearly separated from effects of native species. Specific inclusion criteria were as follows: (1) Co-invasion experiments that included both single-species and multi-species planting treatments of non-native plants, with native species present and maintained at constant composition and abundance across treatments. (2) Experiments using gradients of non-native plant richness in which native species were either absent, or their composition and abundance remained constant across all non-native treatments, allowing non-native diversity effects to be isolated. (3) Studies based on spatial association and removal experiments data in which the performance of non-native species (or native species) was quantified under different invasion scenarios, specifically comparing plots where the focal species co-occurred with a single non-native species versus plots where it co-occurred with two or more non-native species. Differences between these scenarios were used to infer interaction effects among co-invading non-native plants. (4) Plant-soil feedback studies in which soils conditioned by native plants served only as reference (control), whereas soils conditioned by heterospecific non-native species provided the interaction treatment. In addition to extracting data on interactions among non-native plants, we also extracted data on native plant responses experiencing non-native plant interactions (when available). We compared native plant performance under single invasion versus co-invasion scenarios.

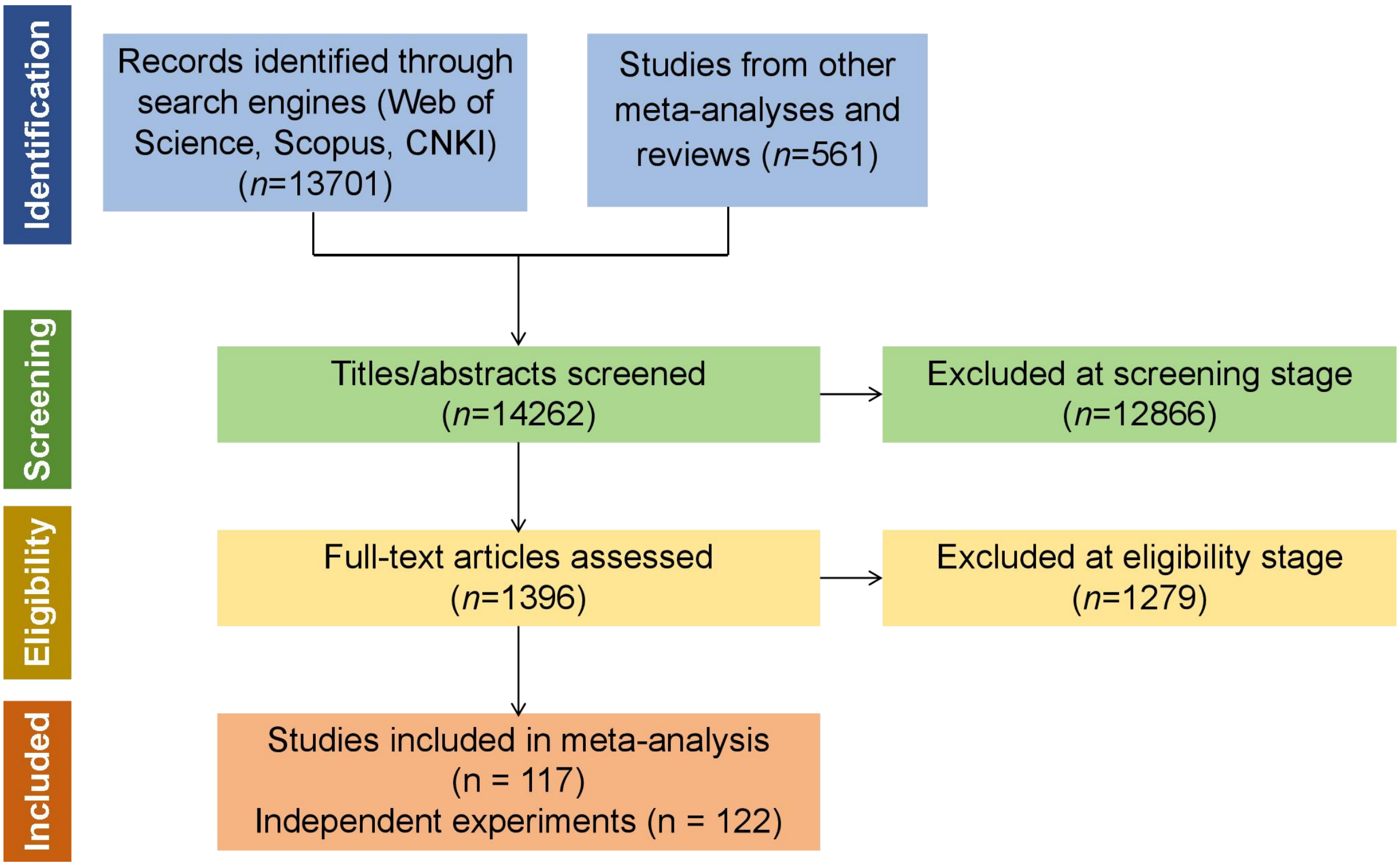

Fig. S1. PRISMA flow diagram for meta-analysis. Flowchart illustrating the processes of study identification, screening, eligibility assessment, and final inclusion in the meta-analysis.

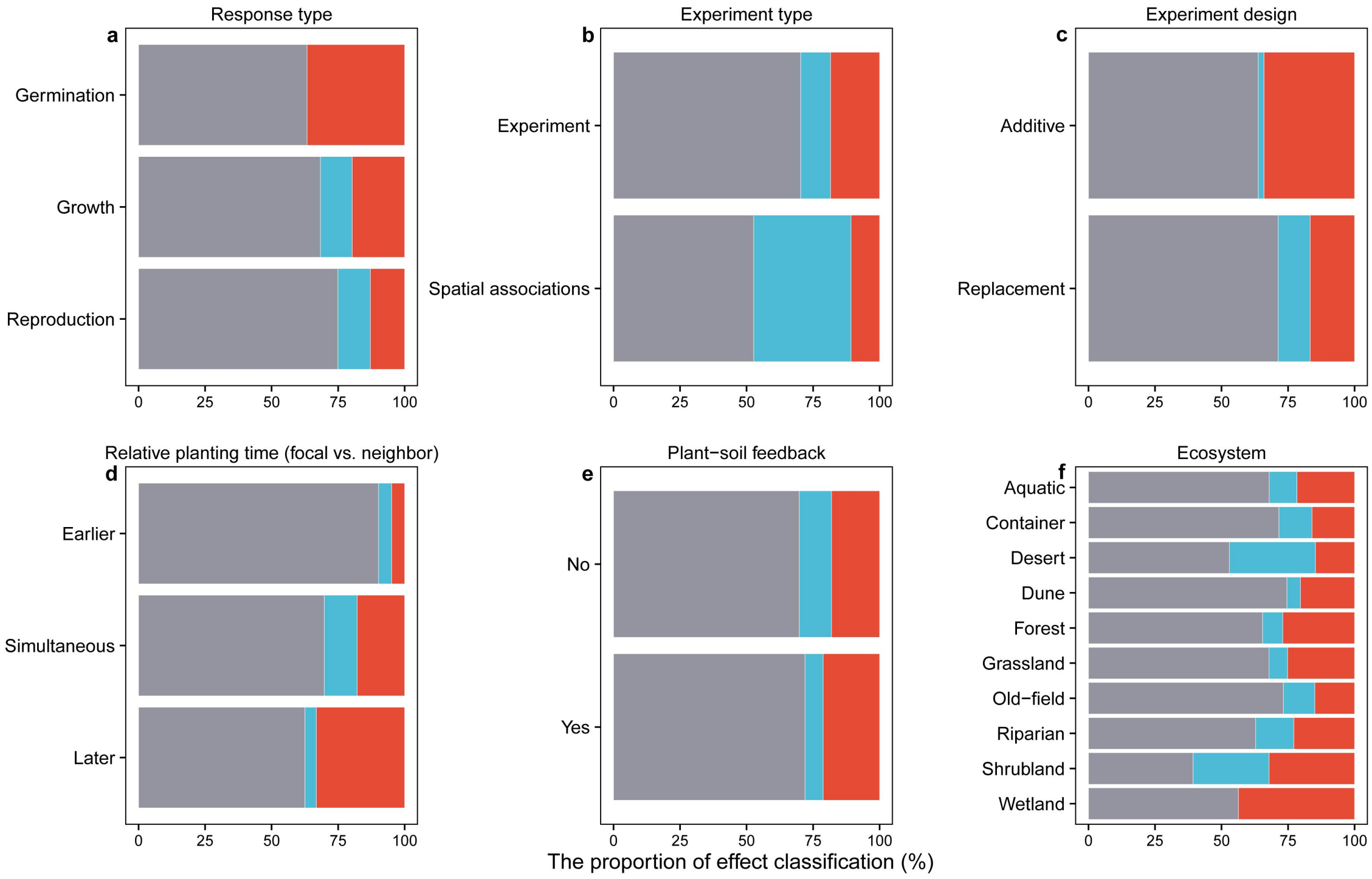

Fig. S2. Effects of experimental context and ecosystem type on the interaction proportions of non-native plants. Study design factors include response type (a), experimental type (b), experimental design (c), planting sequence (d), plant-soil feedback (e) and ecosystem (f). Grey, blue and red represent neutral, promoting and competitive interactions respectively.

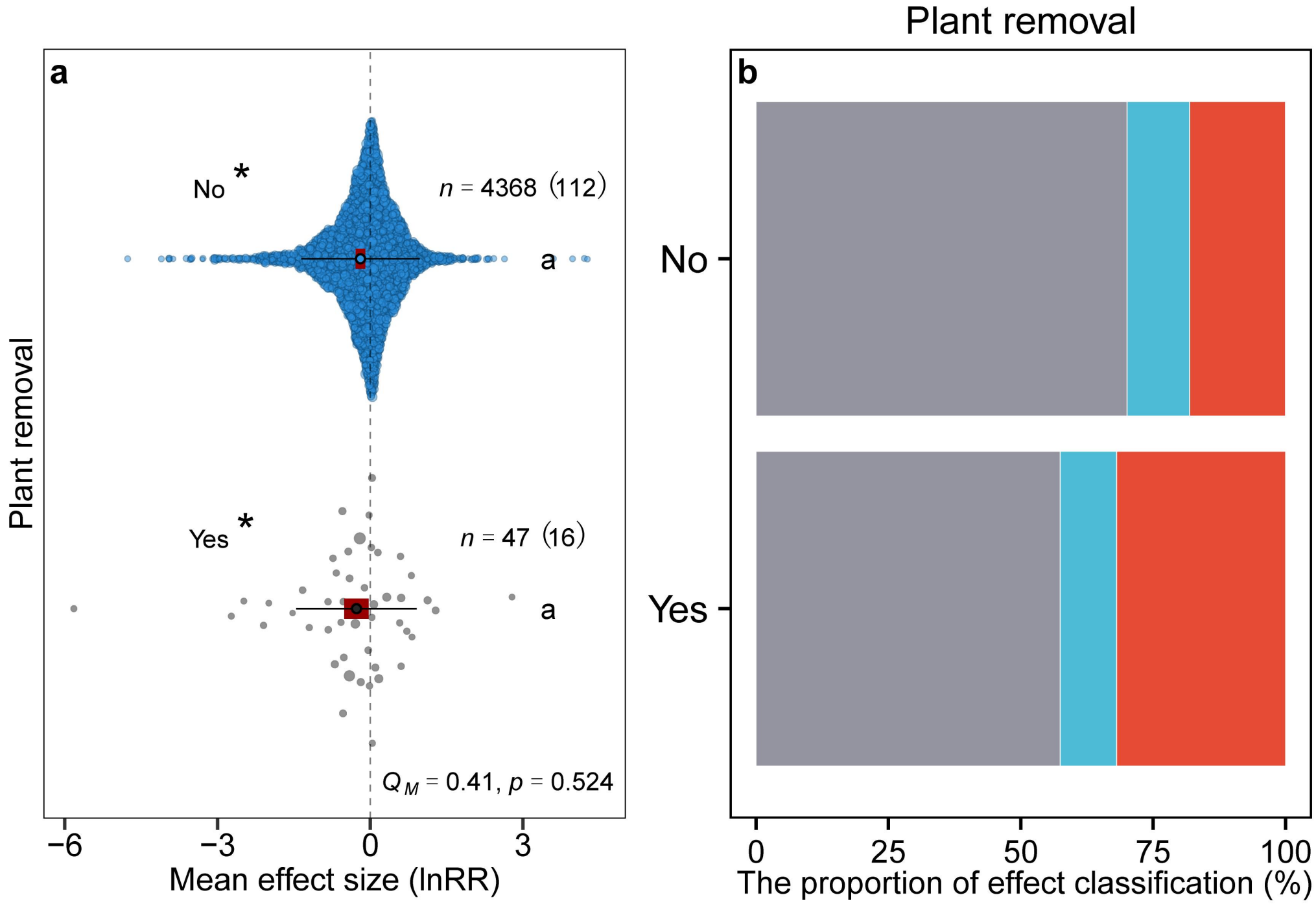

Fig. S3. Effects of plant removal experiments on non-native plant performance (a) and the interaction proportions (b) under co-invasion. Points represent estimated means, while thick red whiskers indicate the 95% confidence intervals and thin black whiskers show 95% prediction intervals from multi-level meta-regression models. Asterisks denote significant effects. *n* represents the number of observations and experiments. Grey, blue and red represent neutral, promoting and competitive interactions respectively.

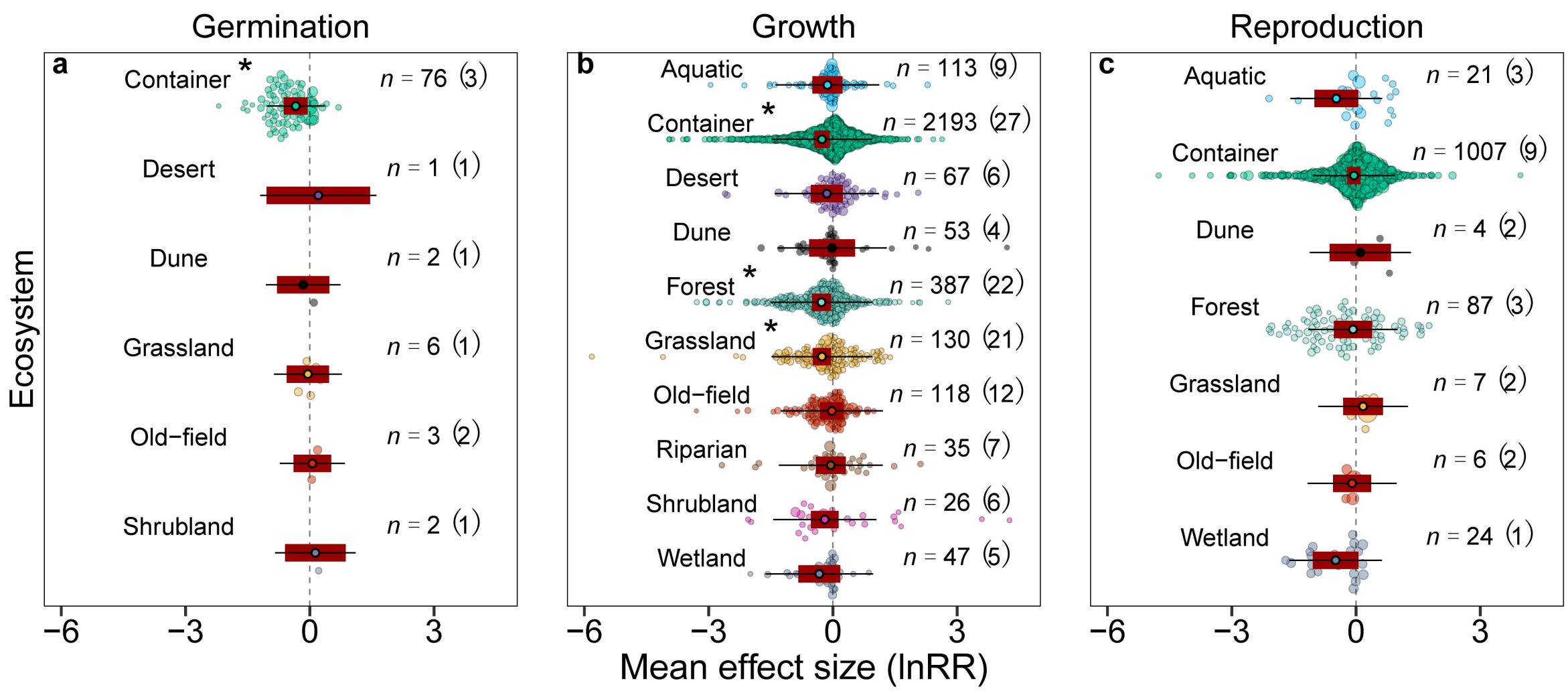

Fig. S4. Different responses of non-native plants to co-invasion by ecosystem. Germination by ecosystem (a), growth by ecosystem (b), reproduction by ecosystem (c). Points represent estimated means, while thick red whiskers indicate the 95% confidence intervals and thin black whiskers show 95% prediction intervals from multi-level meta-regression models. Asterisks denote significant effects. *n* represents the number of observations and experiments.

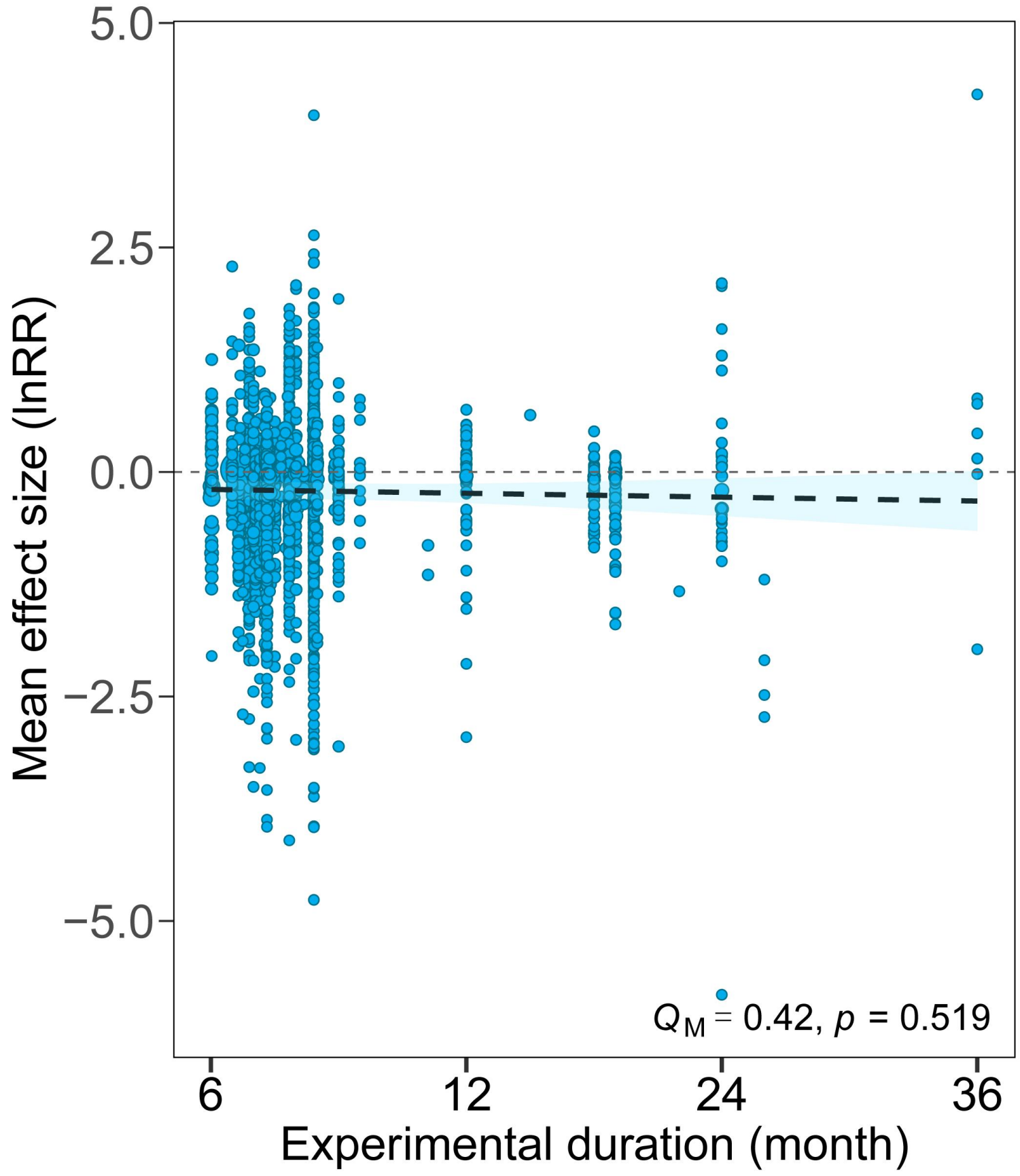

Fig. S5. Relationship between the performance of non-native plants under co-invasion and experimental duration. The size of the points corresponds to the weight of each observation. The regression line indicates the response of the shaded prediction variable at the 95% confidence interval. The values of Q_M and p are displayed in plot. Dashed lines indicate non-significant relationships.

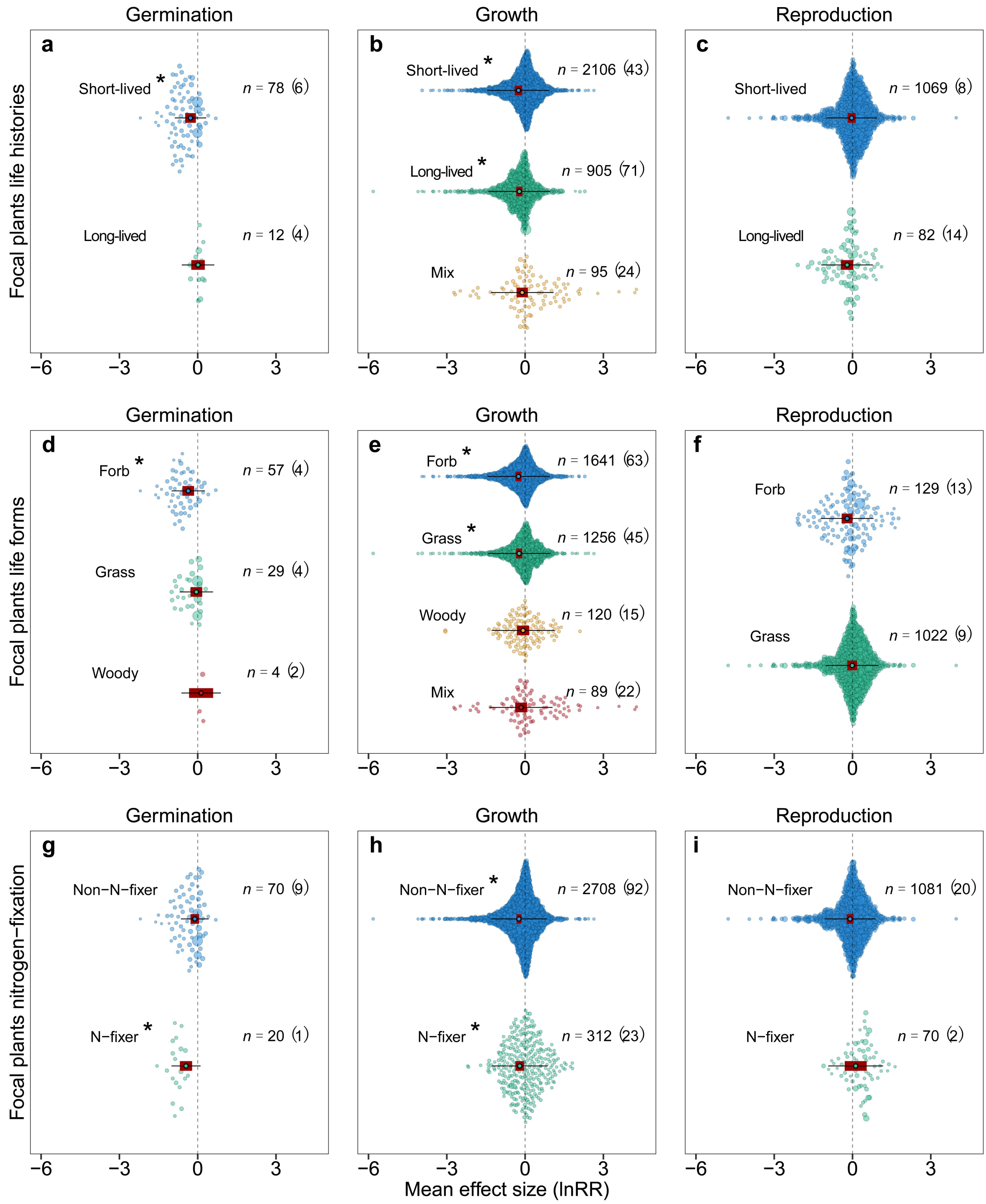

Fig. S6. Different responses of non-native plants to co-invasion by biological traits of focal plants. Germination (a, d, g), growth (b, e, h), reproduction (c, f, i). Points represent estimated means, while thick red whiskers indicate the 95% confidence intervals and thin black whiskers show 95% prediction intervals from multi-level meta-regression models. Asterisks denote significant effects. *n* represents the number of observations and experiments.

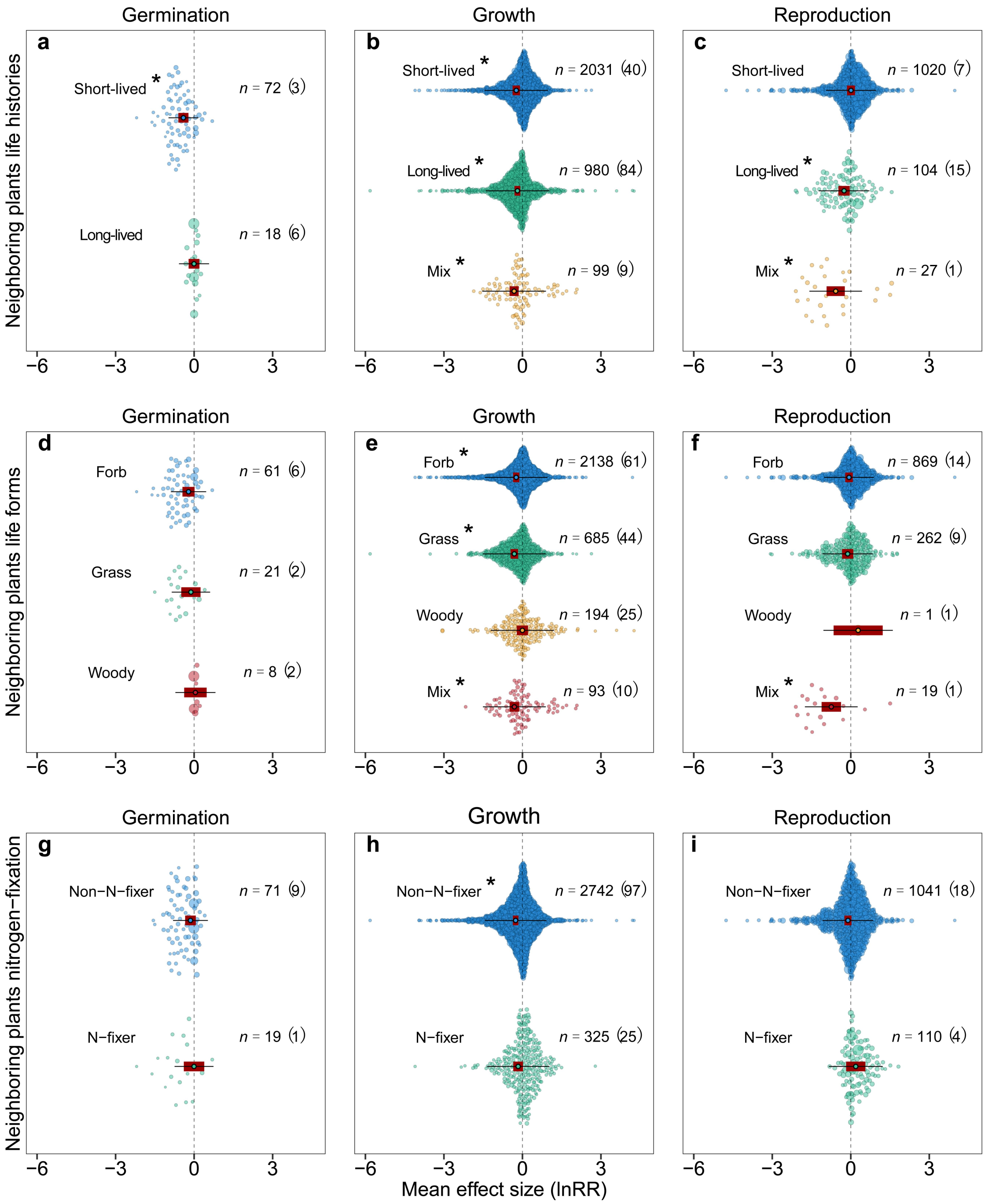

Fig. S7. Different responses of non-native plants to co-invasion by biological traits of neighboring plants. Germination (a, d, g), growth (b, e, h), reproduction (c, f, i). Points represent estimated means, while thick red whiskers indicate the 95% confidence intervals and thin black whiskers show 95% prediction intervals from multi-level meta-regression models. Asterisks denote significant effects. *n* represents the number of observations and experiments.

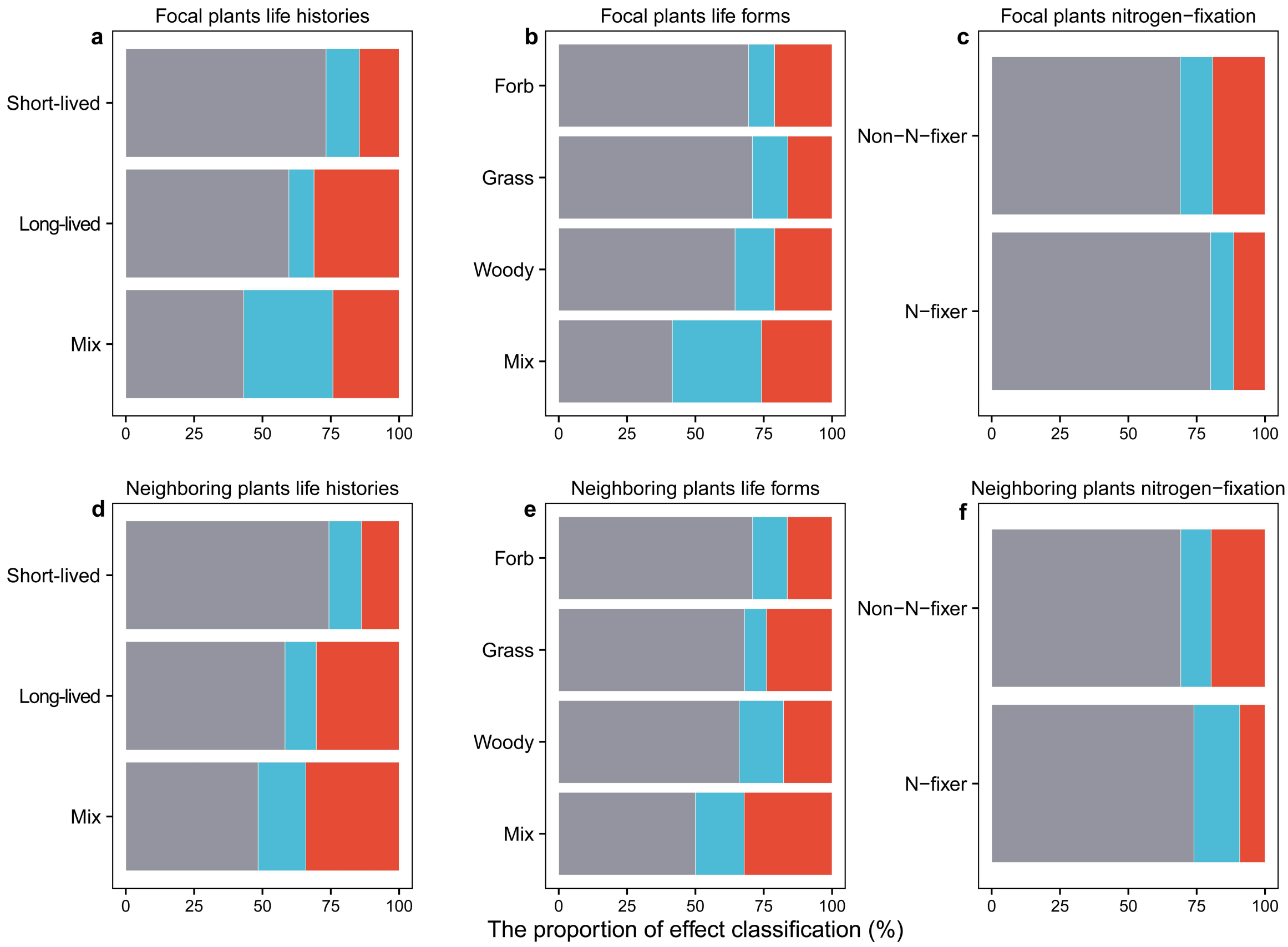

Fig. S8. Effects of biological traits of focal and neighboring plants on the interaction proportions of non-native plants. Biological traits of focal plants (a-c) and neighbouring plants (d-f) are shown. Traits include life histories (a and d), life forms (b and e), and nitrogen-fixing capacity (c and f). Grey, blue and red represent neutral, facilitative and competitive interactions respectively.

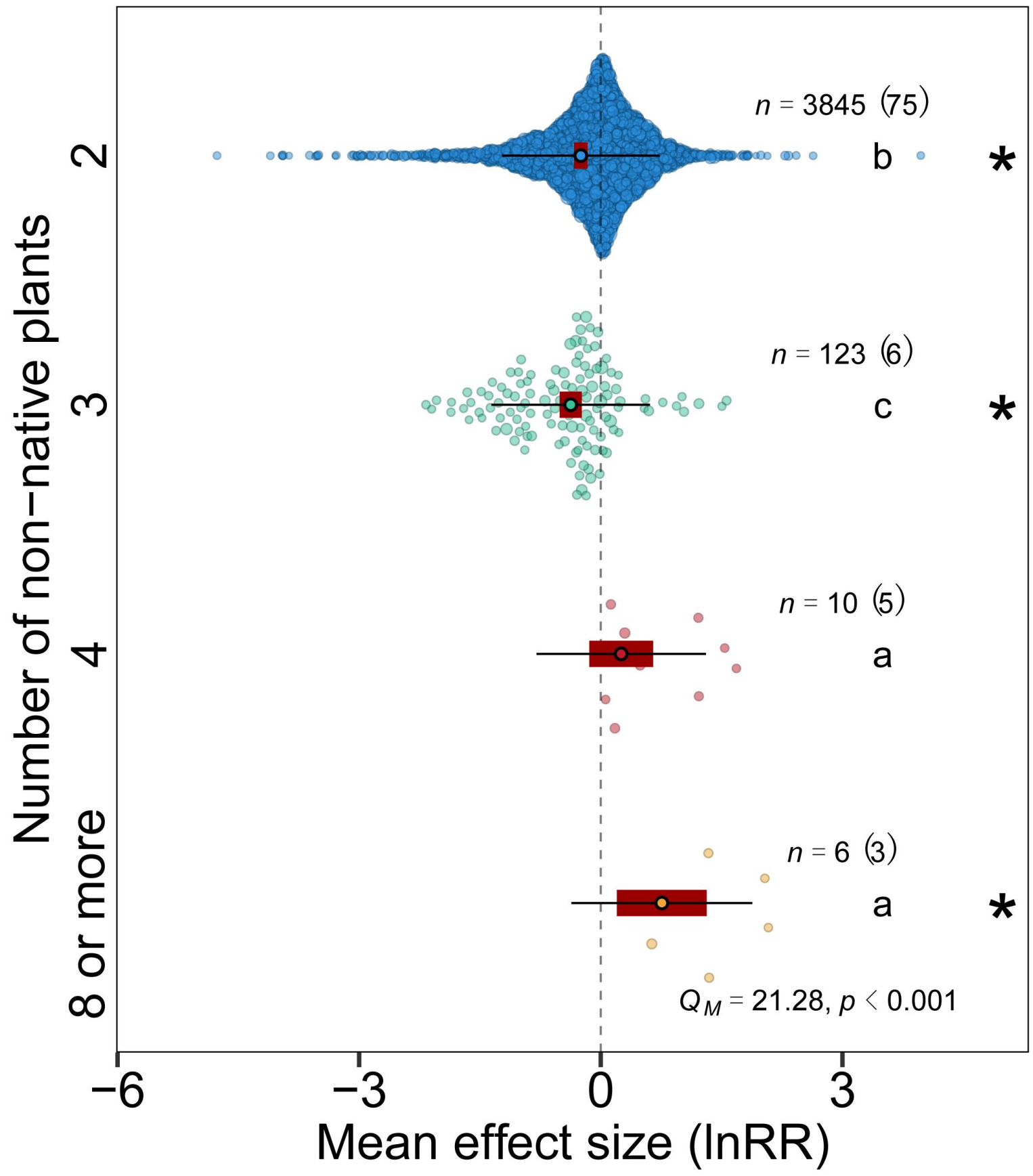

Fig. S9. Effects of non-native plant richness on performance under co-invasion. Orchard plots show the effects of the non-native plants richness on the performance of non-native plants under co-invasion. Points represent estimated means, while thick red whiskers indicate 95% confidence intervals, and thin black whiskers show 95% prediction intervals from multi-level meta-regression models. Asterisks denote significant effects. *n* represents the number of observations and experiments.

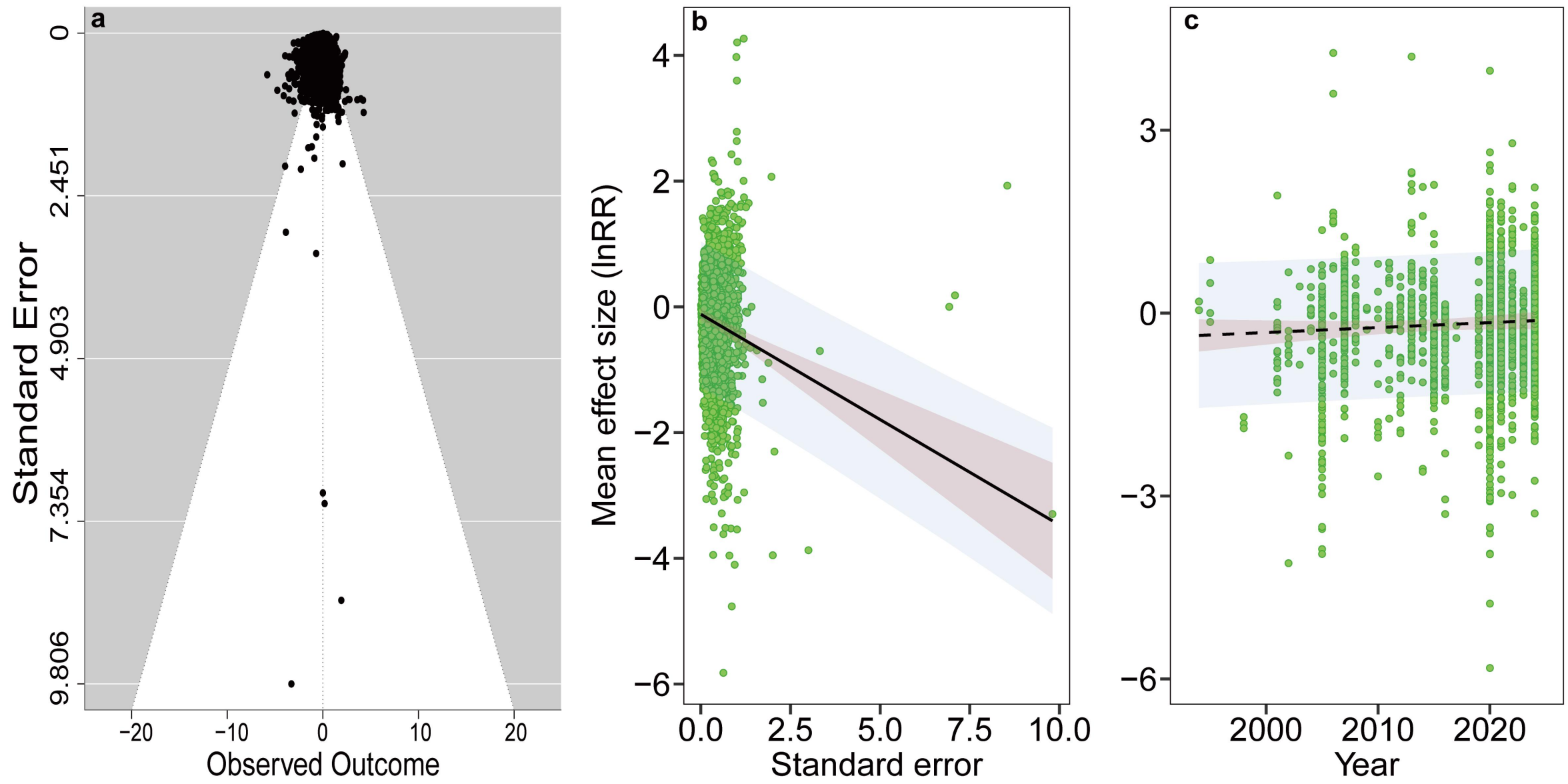

Fig. S10. Assessment of publication bias in the meta-analysis. (a) Funnel plot showing the effect of co-invasion on the performance of non-native plants. (b) Egger's regression test showing the relationship between effect size and standard error. (c) Relationship between effect size and publication year to assess temporal bias. Orange shading represents 95% confidence intervals, blue shading shows 95% prediction intervals and each background point an individual interaction effect size. Solid lines represent significant relationships, while dashed lines indicate non-significant relationships.

Table S1. Distribution of observations and experiments by continent.

Continent	Number of observations	Number of experiments
Africa	5	2
Asia	583	35
Europe	2844	15
Northern America	818	60
Oceania	142	6
Southern America	23	5

Table S2. Distribution of observations and experiments by ecosystem type.

Ecosystem	Number of observations	Number of experiments
Aquatic	134	9
Container	3276	28
Desert	69	6
Dune	59	4
Forest	474	22
Grassland	143	23
Old-field	127	14
Riparian	35	7
Shrubland	28	6
Wetland	71	5

Table S3. Distribution of observations and experiments by experiment type.

Experiment type	Number of observations	Number of experiments
Experiment.field	544	47
Greenhouse	3778	68
Spatial association	93	16

Table S4. Distribution of observations and experiments by experiment design.

Experiment design	Number of observations	Number of experiments
Addition	326	31
Replacement	3640	51

Table S5. Distribution of observations and experiments by non-native plant richness studies.

Non-native richness	Number of observations	Number of experiments
2	3845	75
3	123	6
4	10	5
8 or more	6	3

Table S6. Distribution of observations and experiments by removal experiment status.

Removal experiment	Number of observations	Number of experiments
No	4368	112
Yes	47	16

Table S7. Distribution of observations and experiments by feedback experiment status.

Feedback experiment	Number of observations	Number of experiments
No	4137	107
Yes	278	15

Table S8. The number of observations on non-native plant performance under co-invasion across all experiments.

Response type	Number of observations	Number of experiments
Germination	90	9
Growth	3169	117
Reproduction	1156	22

Table S9. Distribution of interaction direction among co-invading non-native species.

Direction of influence	Number of observations	Percentage
Facilitation	520	11.8%
Neutral	3088	69.9%
Competition	807	18.3%

Table S10. Effects of moderator variables on the performance of non-native plants under co-invasion. Values represent model estimates with 95% confidence intervals (CI; lower and upper) and *p*-values from multilevel meta-regression models.

Moderator variables	Estimate	Lower CI	Upper CI	*p* value
Germination	-0.472	-0.621	-0.323	<0.001
Growth	-0.188	-0.281	-0.094	<0.001
Reproduction	-0.180	-0.279	-0.081	<0.001
Experiment	-0.231	-0.327	-0.135	<0.001
Spatial associations	0.063	-0.173	0.299	0.601
Addition	-0.413	-0.523	-0.302	<0.001
Replacement	-0.217	-0.312	-0.122	<0.001
Removal_No	-0.192	-0.287	-0.098	<0.001
Removal_Yes	-0.270	-0.510	-0.030	0.027
Earlier	0.032	-0.149	0.212	0.731
Simultaneous	-0.191	-0.286	-0.097	<0.001
Later	-0.541	-0.690	-0.393	<0.001
Feedback_No	-0.222	-0.321	-0.123	<0.001
Feedback_Yes	-0.052	-0.285	0.181	0.664
Aquatic	-0.141	-0.495	0.214	0.437
Container	-0.261	-0.435	-0.087	0.003
Desert	-0.152	-0.526	0.223	0.426
Dune	0.032	-0.507	0.571	0.907
Forest	-0.276	-0.502	-0.051	0.016
Grassland	-0.229	-0.436	-0.022	0.030
Old-field	-0.051	-0.307	0.205	0.696
Riparian	-0.047	-0.401	0.306	0.793
Shrubland	-0.186	-0.517	0.144	0.270
Wetland	-0.348	-0.836	0.139	0.162

Table S11. The proportion of interaction types among non-native plants in different experimental contexts and ecosystems.

Moderator variables	Facilitation%	Neutral%	Competition%
Germination	0.0	63.3	36.7
Growth	12.0	68.3	19.7
Reproduction	12.2	74.9	12.9
Experiment	11.2	70.3	18.4
Spatial associations	36.6	52.7	10.8
Addition	2.2	63.8	34.0
Replacement	12.0	71.3	16.7
Removal_No	11.8	70.1	18.1
Removal_Yes	10.6	57.4	31.9
Earlier	4.9	90.2	4.9
Simultaneous	12.3	69.8	17.9
Later	4.3	62.5	33.2
Feedback_NO	12.1	69.8	18.1
Feedback_Yes	6.8	71.9	21.2
Aquatic	10.4	67.9	21.6
Container	12.4	71.6	16.0
Desert	32.4	52.9	14.7
Dune	5.1	74.6	20.3
Forest	7.6	65.4	27.0
Grassland	7.0	67.8	25.2
Old-field	11.8	73.2	15.0
Riparian	14.3	62.9	22.9
Shrubland	28.6	39.3	32.1
Wetland	0.0	56.3	43.7

Table S12. Results of a full mixed-effects meta-analysis model simultaneously testing multiple moderator variables (experimental contexts) affecting non-native plant performance. Values represent model estimates with 95% confidence intervals (CI; lower and upper) and *p*-values from multilevel meta-regression models.

Moderator variables	Estimate	Lower CI	Upper CI
Germination	-0.566	-0.749	-0.383
Growth	-0.283	-0.426	-0.140
Reproduction	-0.275	-0.421	-0.128
Experiment	-0.294	-0.438	-0.150
Spatial associations	0.051	-0.261	0.363
Removal_No	-0.287	-0.430	-0.143
Removal_Yes	-0.279	-0.566	0.009
Earlier	-0.041	-0.251	0.169
Simultaneous	-0.276	-0.419	-0.133
Later	-0.618	-0.804	-0.433
Feedback_No	-0.303	-0.452	-0.155
Feedback_Yes	-0.039	-0.304	0.227
Aquatic	-0.141	-0.502	0.220
Container	-0.294	-0.477	-0.111
Desert	-0.305	-0.706	0.097
Dune	0.030	-0.526	0.586
Forest	-0.385	-0.636	-0.134
Grassland	-0.229	-0.445	-0.013
Old-field	-0.058	-0.323	0.207
Riparian	-0.222	-0.618	0.174
Shrubland	-0.282	-0.624	0.060
Wetland	-0.387	-0.890	0.115

Table S13. Effects of focal plant biological traits on non-native plant performance under co-invasion. Values represent model estimates with 95% confidence intervals (CI; lower and upper) and *p*-values from multilevel meta-regression models.

**Biological traits**	**Estimate**	**Lower CI**	**Upper CI**	***p* value**
Short-lived	-0.248	-0.386	-0.111	<0.001
Long-lived	-0.215	-0.330	-0.100	<0.001
Mixed-histories	-0.103	-0.314	0.108	0.339
Forb	-0.244	-0.353	-0.134	<0.001
Grass	-0.228	-0.340	-0.117	<0.001
Woody	-0.081	-0.305	0.143	0.478
Mixed-forms	-0.146	-0.368	0.076	0.198
Non-nitrogen-fixing	-0.229	-0.318	-0.140	<0.001
Nitrogen-fixing	-0.201	-0.364	-0.038	0.016

Table S14. Effects of neighboring plant biological traits on non-native plant performance under co-invasion. Values represent model estimates with 95% confidence intervals (CI; lower and upper) and *p*-values from multilevel meta-regression models.

**Biological traits**	**Estimate**	**Lower CI**	**Upper CI**	***p* value**
Short-lived	-0.229	-0.368	-0.090	0.001
Long-lived	-0.181	-0.289	-0.074	0.001
Mixed-histories	-0.346	-0.507	-0.185	<0.001
Forb	-0.221	-0.337	-0.106	<0.001
Grass	-0.298	-0.441	-0.156	<0.001
Woody	-0.002	-0.212	0.207	0.984
Mixed-forms	-0.367	-0.537	-0.196	<0.001
Non-nitrogen-fixing	-0.255	-0.355	-0.155	<0.001
Nitrogen-fixing	-0.149	-0.335	0.036	0.115

Table S15. The proportion of interaction types among focal non-native plants in different biological traits.

**Biological traits**	**Facilitation%**	**Neutral%**	**Competition%**
Short-lived	12.2	73.3	14.5
Long-lived	9.2	59.7	34.1
Mixed-histories	32.6	43.2	24..2
Forb	9.5	69.5	21.0
Grass	13.0	70.8	16.2
Woody	14.5	64.5	21.0
Mixed-forms	32.6	41.6	25.8
Non-nitrogen-fixing	11.9	69.0	19.1
Nitrogen-fixing	8.5	80.1	11.4

Table S16. The proportion of interaction types among neighboring non-native plants in different biological traits.

Biological traits	Facilitation%	Neutral%	Competition%
Short-lived	11.9	74.3	13.8
Long-lived	11.4	58.3	30.3
Mixed-histories	17.5	48.4	34.1
Forbs	12.7	70.9	16.4
Grasser	8.1	68.0	24.0
Woody	16.3	66.0	17.7
Mixed-forms	17.9	50.0	32.1
Non-nitrogen-fixing	11.1	69.2	19.7
Nitrogen-fixing	16.7	74.0	9.3

Table S17. Results of a full mixed-effects meta-analysis model simultaneously testing multiple moderator variables (biological traits) affecting non-native plant performance. Values represent model estimates with 95% confidence intervals (CI; lower and upper) and *p*-values from multilevel meta-regression models.

	**Moderator variables**	**Estimate**	**Lower CI**	**Upper CI**
Focal plants	Short-lived	-0.261	-0.399	-0.122
	Long-lived	-0.258	-0.385	-0.131
	Mixed-histories	0.094	-0.823	1.010
	Forbs	-0.271	-0.395	-0.147
	Grasser	-0.258	-0.385	-0.131
	Woody	-0.132	-0.409	0.145
	Mixed-forms	0.626	-0.814	2.067
	Non-nitrogen-fixing	-0.260	-0.383	-0.137
	Nitrogen-fixing	-0.253	-0.450	-0.056
Neighboring plants	Short-lived	-0.250	-0.390	-0.109
	Long-lived	-0.274	-0.404	-0.144
	Mixed-histories	-0.447	-0.662	-0.232
	Forbs	-0.246	-0.376	-0.116
	Grasser	-0.298	-0.458	-0.138
	Woody	-0.221	-0.523	0.082
	Mixed-forms	-0.413	-0.642	-0.184
	Non-nitrogen-fixing	-0.264	-0.388	-0.140
	Nitrogen-fixing	-0.219	-0.433	-0.005

Table S18. Effects of trait combinations of focal and neighboring plant on non-native plant performance under co-invasion. Values represent model estimates with 95% confidence intervals (CI; lower and upper) and *p*-values from multilevel meta-regression models.

**Biological traits**	**Estimate**	**Lower CI**	**Upper CI**	***p* value**
Short-lived on short-lived	-0.281	-0.432	-0.129	<0.001
Short-lived on long-lived	-0.224	-0.395	-0.052	0.011
Long-lived on short-lived	-0.231	-0.379	-0.082	0.002
Long-lived on long-lived	-0.249	-0.363	-0.135	<0.001
Forb on forb	-0.261	-0.369	-0.153	<0.001
Forb on grass	-0.241	-0.353	-0.129	<0.001
Forb on woody	-0.491	-1.251	0.269	0.206
Grass on forb	-0.322	-0.459	-0.185	<0.001
Grass on grass	-0.334	-0.474	-0.194	<0.001
Grass on woody	-0.374	-0.811	0.063	0.094
Woody on forb	-0.197	-0.537	0.143	0.257
Woody on grass	-0.214	-0.611	0.183	0.292
Woody on woody	0.022	-0.222	0.266	0.862

Table S19. Effects of non-native plant richness on alien plant performance under co-invasion. Values represent model estimates with 95% confidence intervals (CI; lower and upper) and *p*-values from multilevel meta-regression models.

Non-native richness	Estimate	Lower CI	Upper CI	*p* value
Richness2	-0.248	-0.331	-0.165	<0.001
Richness3	-0.374	-0.511	-0.236	<0.001
Richness4	0.253	-0.142	0.649	0.209
Richness8 or more	0.758	0.200	1.315	0.008

List of studies included in our database

Adler PB, D'Antonio CM, Tunison JTJPS. 1998. Understory Succession Following a Dieback of Myrica faya in Hawai'i Volcanoes National Park. 52.

Ahmad R, Rashid I, Hamid M, Malik AH, Khuroo AA. 2021. Invasion shadows in soil system overshadow the restoration of invaded ecosystems: implications for invasive plant management. Ecological Engineering 164: 106219.

Allen WJ, Wainer R, Tylianakis JM, Barratt BIP, Shadbolt M-R, Waller LP, Dickie IA. 2020. Community-level direct and indirect impacts of an invasive plant favour exotic over native species. Journal of Ecology 108: 2499-2510.

Alvarez ME, Cushman JH. 2002. Community-level consequences of a plant invasion: Effects on three habitats in coastal California. Ecological Applications 12: 1434-1444.

Barrat-Segretain MH, Elger A. 2004. Experiments on growth interactions between two invasive macrophyte species. Journal of Vegetation Science 15: 109-114.

Belote RT, Weltzin JF. 2006. Interactions between two co-dominant, invasive plants in the understory of a temperate deciduous forest. Biological Invasions 8: 1629-1641.

Besaw LM, Thelen GC, Sutherland S, Metlen K, Callaway RM. 2011. Disturbance, resource pulses and invasion: short-term shifts in competitive effects, not growth responses, favour exotic annuals. Journal of Applied Ecology 48: 998-1006.

Brigham LM, Coffey J, Lulow ME, Ta P, Kimball S. 2024. Persistent invasion by non-native species and transition to an upland community after removal of invasive Tamarix in a Californian cienega. Journal of Arid Environments 224.

Callaway RM, Mahall BE, Wicks C, Pankey J, Zabinski C. 2003. Soil fungi and the effects of an invasive forb on grasses: Neighbor identity matters. Ecology 84: 129-135.

Cao Y, Wang T, Xiao Ya, Zhou B. 2014. The interspecific competition between Humulus scandens and Alternanthera philoxeroides. Journal of Plant Interactions 9: 194-199.

Carino DA, Daehler CC. 2002. Can inconspicuous legumes facilitate alien grass invasions? Partridge peas and fountain grass in Hawai'i. Ecography 25: 33-41.

Chen D, van Kleunen M. 2022. Invasional meltdown mediated by plant–soil feedbacks may depend on community diversity. New Phytologist 235: 1589-1598.

Chen D, van Kleunen M. 2024. Negative conspecific plant-soil feedback on alien plants co-growing with natives is partly mitigated by another alien. Plant and Soil.

Cleland EE, Esch E, McKinney J. 2015. Priority effects vary with species identity and origin in an experiment varying the timing of seed arrival. Oikos 124: 33-40.

Cook-Patton SC, Agrawal AA. 2014. Exotic plants contribute positively to biodiversity functions but reduce native seed production and arthropod richness. Ecology 95: 1642-1650.

Cushman JH, Gaffney KA. 2010. Community-level consequences of invasion: impacts of exotic clonal plants on riparian vegetation. Biological Invasions 12: 2765-2776.

Cushman JH, Lortie CJ, Christian CE. 2011. Native herbivores and plant facilitation mediate the performance and distribution of an invasive exotic grass. Journal of Ecology 99: 524-531.

Dai W, Ni X, Zhao C. 2024. Soil Legacy Effects on Seedling Growth and Competition of Two Invasive Alien Plants: Chromolaena odorata and Bidens pilosa. Research of Environmental Sciences 37: 2278-2287.

D'Antonio CM, Hughes RF, Vitousek PM. 2001. Factors influencing dynamics of two invasive C4 grasses in seasonally dry Hawaiian woodlands. Ecology 82: 89-104.

Davies KW, Nafus AM, Sheley RL. 2010. Non-native competitive perennial grass impedes the spread of an invasive annual grass. Biological Invasions 12: 3187-3194.

Dodd LL, Harms NE, Schad AN. 2021. Reciprocal competitive effects of congeneric invaders, Trapa natans L. and Trapa bispinosa Roxb. var. iinumai Nakano, in established freshwater plant cultures. Aquatic Botany 174.

Dubrovin DI, Veselkin DV, Gusev AP. 2023. Plant Species Richness and Invasional Meltdown in Different Parts of Acer negundo L. Secondary Range. Forests 14.

Dukes JS. 2001. Biodiversity and invasibility in grassland microcosms. Oecologia 126: 563-568.

Emery SM, Doran PJ, Legge JT, Kleitch M, Howard S. 2013. Aboveground and belowground impacts following removal of the invasive species baby's breath (Gypsophila paniculata) on Lake Michigan sand dunes. Restoration Ecology 21: 506-514.

Engel EC, Weltzin JF. 2008. Can community composition be predicted from pairwise species interactions? Plant Ecology 195: 77-85.

Facelli JMJE. 1994. Multiple indirect effects of plant litter affect the establishment of woody

seedlings in old fields. 75: 1727-1735.

Fang X-H, Chen X-M, Liu R-L, Zhang S-H, Gao J-Q, Dong B-C, Yu F-H. 2024. Effects of alligator weed invasion on wetlands in protected areas: A case study of Lishui Jiulong National Wetland Park. Science of the Total Environment 953.

Ferenc V, Merkert C, Zilles F, Sheppard CS. 2021. Native and alien species suffer from late arrival, while negative effects of multiple alien species on natives vary. Oecologia 197: 271-281.

Ferenc V, Sheppard CS. 2020. The stronger, the better - trait hierarchy is driving alien species interaction. Oikos 129: 1455-1467.

Figiel Jr CR, Collins B, Wein GJBotTBC. 1995. Variation in survival and biomass of two wetland grasses at different nutrient and water levels over a six week period. 24-29.

Flory SL, Bauer JT. 2014. Experimental evidence for indirect facilitation among invasive plants. Journal of Ecology 102: 12-18.

Fortner AM, Weltzin JF. 2007. Competitive hierarchy for four common old-field plant species depends on resource identity and availability. Journal of the Torrey Botanical Society 134: 166-176.

Garcia-Serrana H, Sans FX, Escarre J. 2007. Interspecific competition between alien and native congeneric species. Acta Oecologica-International Journal of Ecology 31: 69-78.

Graebner RC, Callaway RM, Montesinos D. 2012. Invasive species grows faster, competes better, and shows greater evolution toward increased seed size and growth than exotic non-invasive congeners. Plant Ecology 213: 545-553.

Grove S, Parker IM, Haubensak KA. 2015. Persistence of a soil legacy following removal of a nitrogen-fixing invader. Biological Invasions 17: 2621-2631.

Haling RE, Campbell CD, Tighe MK, Guppy CN. 2013. Effect of competition from a C4 grass on the phosphorus response of a subtropical legume. Crop & Pasture Science 64: 985-992.

He Z, Zhang X, Fan S, Chen R, Sun J, Ma F, Zhao P, Liu J, Zhang H, Liu C. 2024. Effects of the interspecific competition between Eichhornia crassipes and Alternanthera philoxeroides and the additional impact of their biological control agents: relevance for aquatic weed management. Hydrobiologia.

Hulme PE, Bremner ET. 2006. Assessing the impact of Impatiens glandulifera on riparian habitats:: partitioning diversity components following species removal. Journal of Applied Ecology 43: 43-50.

Isbell FI, Wilsey BJ. 2011. Increasing native, but not exotic, biodiversity increases aboveground productivity in ungrazed and intensely grazed grasslands. Oecologia 165: 771-781.

Jordan NR, Larson DL, Huerd SC. 2008. Soil modification by invasive plants: effects on native and invasive species of mixed-grass prairies. Biological Invasions 10: 177-190.

Kawaletz H, Moelder I, Zerbe S, Annighoefer P, Terwei A, Ammer C. 2013. Exotic tree seedlings are much more competitive than natives but show underyielding when growing together. Journal of Plant Ecology 6: 305-315.

Khan N, George D, Shabbir A, Hanif Z, Adkins SW. 2015. Rising CO_2 can alter fodder-weed interactions and suppression of Parthenium hysterophorus. Weed Research 55: 113-117.

Kinlock NL. 2021. Uncovering structural features that underlie coexistence in an invaded woody plant community with interaction networks at multiple life stages. Journal of Ecology 109: 384-398.

Kivlin SN, Hawkes CV. 2011. Differentiating between effects of invasion and diversity: impacts of aboveground plant communities on belowground fungal communities. New Phytologist 189: 526-535.

Kuebbing SE, Classen AT, Call JJ, Henning JA, Simberloff D. 2015. Plant-soil interactions promote co-occurrence of three nonnative woody shrubs. Ecology 96: 2289-2299.

Kuebbing SE, Classen AT, Sanders NJ, Simberloff D. 2015. Above- and below-ground effects of plant diversity depend on species origin: an experimental test with multiple invaders. New Phytologist 208: 727-735.

Kuebbing SE, Classen AT, Simberloff D. 2014. Two co-occurring invasive woody shrubs alter soil properties and promote subdominant invasive species. Journal of Applied Ecology 51: 124-133.

Kuebbing SE, Patterson CM, Classen AT, Simberloff D. 2016. Co-occurring nonnative woody shrubs have additive and non-additive soil legacies. Ecological Applications 26: 1896-1906.

Kueffer C. 2010. Reduced risk for positive soil-feedback on seedling regeneration by invasive trees on a very nutrient-poor soil in Seychelles. Biological Invasions 12: 97-102.

Lanta V, Liancourt P, Altman J, Cerny T, Dvorsky M, Fibich P, Gotzenberger L, Hornych O, Miklin J, Petrik P, et al. 2022. Determinants of invasion by single versus multiple plant species in temperate lowland forests. Biological Invasions 24: 2513-2528.

Leicht-Young SA, Bois ST, Silander JA, Jr. 2015. Impacts of Celastrus-primed soil on common native and invasive woodland species. Plant Ecology 216: 503-516.

Li Y, Xu X. 2024. No evidence that modification of soil microbiota by woody invader facilitates subsequent invasion by herbaceous species. Ecological Applications 34: e2807.

Magnoli SM, Kleinhesselink AR, Cushman JH. 2013. Responses to invasion and invader removal differ between native and exotic plant groups in a coastal dune. Oecologia 173: 1521-1530.

Mangla S, Sheley RL, James JJ, Radosevich SR. 2011. Intra and interspecific competition among invasive and native species during early stages of plant growth. Plant Ecology 212: 531-542.

Metlen KL, Aschehoug ET, Callaway RM. 2013. Competitive outcomes between two exotic invaders are modified by direct and indirect effects of a native conifer. Oikos 122: 632-640.

Molina-Montenegro MA, Badano EI, Cavieres LA. 2008. Positive interactions among plant species for pollinator service: assessing the 'magnet species' concept with invasive species. Oikos 117: 1833-1839.

Mony C, Koschnick TJ, Haller WT, Muller S. 2007. Competition between two invasive Hydrocharitaceae (*Hydrilla verticillata* (L.f.) (Royle) and *Egeria densa* (Planch)) as influenced by sediment fertility and season. Aquatic Botany 86: 236-242.

Mostow RS, Barreto FS, Hacker SD. 2024. A hybrid beachgrass (*Ammophila arenaria* x *A. breviligulata*) is more productive and outcompetes its non-native parent species. Oecologia 205: 81-94.

Nielsen JA, Frew RD, Whigam PA, Callaway RM, Dickinson KJM. 2015. Germination and growth responses of co-occurring grass species to soil from under invasive Thymus vulgaris. Allelopathy Journal 35: 139-152.

Oduor AMO, Yu H, Liu Y. 2024. Invasive plant species support each other's growth in

low-nutrient conditions but compete when nutrients are abundant. Ecology 105.

Ortega YK, Pearson DE. 2010. Effects of Picloram Application on Community Dominants Vary With Initial Levels of Spotted Knapweed (Centaurea stoebe) Invasion. Invasive Plant Science and Management 3: 70-80.

Oschrin E, Reynolds HL. 2019. Co-occurring invasive plant interactions do not predict the impacts of invasion in experimental tallgrass prairie communities. Biological Invasions 21: 2417-2430.

Oschrin E, Reynolds HL. 2020. Interpreting Pattern in Plant-Soil Feedback Experiments with Co-occurring Invasive Species: A Graphical Framework and Case Study. Diversity-Basel 12.

Paulus KR, Marshall JM. 2022. Competitive Interactions between Two Non-Native Species (Alliaria petiolata M. Bieb. Cavara & Grande and Hesperis matronalis L.) and a Native Species (Ageratina altissima L. R.M. King & H. Rob.). Plants-Basel 11.

Pfeifer-Meister L, Cole EM, Roy BA, Bridgham SD. 2008. Abiotic constraints on the competitive ability of exotic and native grasses in a Pacific Northwest prairie. Oecologia 155: 357-366.

Poon GT, Maherali H. 2015. Competitive interactions between a nonmycorrhizal invasive plant, Alliaria petiolata, and a suite of mycorrhizal grassland, old field, and forest species. PeerJ 3.

Puliafico KP, Schwarzlaender M, Price WJ, Harmon BL, Hinz HL. 2011. Native and Exotic Grass Competition with Invasive Hoary Cress (*Cardaria draba*). Invasive Plant Science and Management 4: 38-49.

Rauschert ESJ, Shea K. 2012. Invasional interference due to similar inter- and intraspecific competition between invaders may affect management. Ecological Applications 22: 1413-1420.

Reeb RA, Kuebbing SE. 2024. Phenology mediates direct and indirect interactions among co-occurring invasive plant species. Ecology.

Ren J, Chen P, Shen C, Tao Z, Huang W. 2023. Functional and phylogenetic similarities of co-occurring invaders affect the growth of an invasive forb. Journal of Plant Ecology 16: rtad007.

Rice KJ, Dyer AR. 2001. Seed aging, delayed germination and reduced competitive ability in

Bromus tectorum. Plant Ecology 155: 237-243.

Rodriguez-Echeverria S, Afonso C, Correia M, Lorenzo P, Roiloa SR. 2013. The effect of soil legacy on competition and invasion by Acacia dealbata Link. Plant Ecology 214: 1139-1146.

Rogers WE, Siemann E. 2004. Invasive ecotypes tolerate herbivory more effectively than native ecotypes of the Chinese tallow tree Sapium sebiferum. Journal of Applied Ecology 41: 561-570.

Ruwanza S, Gaertner M, Esler KJ, Richardson DM. 2013. The effectiveness of active and passive restoration on recovery of indigenous vegetation in riparian zones in the Western Cape, South Africa: A preliminary assessment. South African Journal of Botany 88: 132-141.

Shi B, Osunkoya OOO, Soni A, Campbell S, Dhileepan K. 2023. Growth of the invasive Navua sedge (*Cyperus aromaticus*) under competitive interaction with pasture species and simulated grazing conditions: Implication for management. Ecological Research 38: 331-346.

Skalova H, Jarosik V, Dvorackova S, Pysek P. 2013. Effect of Intra- and Interspecific Competition on the Performance of Native and Invasive Species of Impatiens under Varying Levels of Shade and Moisture. PLoS ONE 8.

Skurski TC, Maxwell BD, Rew LJ. 2013. Ecological tradeoffs in non-native plant management. Biological Conservation 159: 292-302.

Slesak RA, Harrington TB, D'Amato AW, Peter DH. 2022. Removal of invasive Scotch broom increases its negative effects on soil chemistry and plant communities. Oecologia 198: 243-254.

Stanescu S, Maherali H. 2017. Arbuscular mycorrhizal fungi alter the competitive hierarchy among old-field plant species. Oecologia 183: 479-491.

Stevens JM, Fehmi JS. 2009. Competitive Effect of Two Nonnative Grasses on a Native Grass in Southern Arizona. Invasive Plant Science and Management 2: 379-385.

Takakura K-I, Fujii S. 2010. Reproductive interference and salinity tolerance differentiate habitat use between two alien cockleburs: *Xanthium occidentale* and *X. italicum* (Compositae). Plant Ecology 206: 309-319.

Tecco PA, Diaz S, Gurvich DE, Perez-Harguindeguy N, Cabido M, Bertone GA. 2007. Facilitation and interference underlying the association between the woody invaders Pyracantha angustifolia and Ligustrum lucidum. Applied Vegetation Science 10: 211-218.

Tecco PA, Gurvich DE, Díaz S, Pérez-Harguindeguy NP, Cabido M. 2006. Positive interaction between invasive plants:: The influence of Pyracantha angustifolia on the recruitment of native and exotic woody species. Austral Ecology 31: 293-300.

Tekiela DR, Barney JN. 2017. Co-invasion of similar invaders results in analogous ecological impact niches and no synergies. Biological Invasions 19: 147-159.

Thouvenot L, Puech C, Martinez L, Haury J, Thiebaut G. 2013. Strategies of the invasive macrophyte Ludwigia grandiflora in its introduced range: competition, facilitation or coexistence with native and exotic species? Aquatic Botany 107: 8-16.

Torres A, Moran-Lopez T, Rodriguez-Cabal MAA, Nunez MA. 2023. Timing of invasive species removal influences nonnative biotic resistance and trajectories of community reassembly. Journal of Ecology 111: 2342-2356.

Uddin MN, Asaeda T, Shampa SH, Robinson RW. 2020. Allelopathy and its coevolutionary implications between native and non-native neighbors of invasive Cynara cardunculus L. Ecology and Evolution 10: 7463-7475.

Vink SN, Jordan NR, Huerd SC, Shaeffer CC, Kinkel LL, Aldrich-Wolfe L. 2015. Soil conditioning effects of native and exotic grassland perennials on the establishment of native and exotic plants. Plant and Soil 393: 335-349.

Vujanovic D, Losapio G, Milic S, Milic D. 2022. The Impact of Multiple Species Invasion on Soil and Plant Communities Increases With Invasive Species Co-occurrence. Frontiers in Plant Science 13.

Wang C, Wei M, Wang S, Wu B, Cheng H. 2020. Erigeron annuus (L.) Pers. and Solidago canadensis L. antagonistically affect community stability and community invasibility under the co-invasion condition. Science of the Total Environment 716.

Wang T, Han J, Fang H, Khan AA, Tang L, Zhang M, Shi F. 2021. The enhanced functional traits contribute to the successful invasion of *Amaranthus palmeri* in salinity environments: a comparison with its congeners. Biologia 76: 2455-2465.

Wang X, Wang J, Hu B, Zheng W-L, Li M, Shen Z-X, Yu F-H, Schmid B, Li M-H. 2022. Richness, not evenness, of invasive plant species promotes invasion success into native plant communities via selection effects. Oikos 2022: e08966.

White VA, Holt JS. 2005. Competition of artichoke thistle (Cynara cardunculus) with native

and exotic grassland species. Weed Science 53: 826-833.

Wilsey B, Martin L, Xu X, Isbell F, Polley HW. 2024. Biodiversity: Net primary productivity relationships are eliminated by invasive species dominance. Ecology Letters 27.

Wilsey BJ, Daneshgar PP, Hofmockel K, Polley HW. 2014. Invaded grassland communities have altered stability-maintenance mechanisms but equal stability compared to native communities. Ecology Letters 17: 92-100.

Woo S, Kim T-M, Kim Y, Jeong S, Kim E. 2024. Inter- and Intraspecific Competition in Invasive Lactuca serriola and Co-Occurring Weedy Plant Species. Ecology and Evolution 14.

Wu H, Dong S, Wang L, Zhu Y, Jia S, Rao B. 2023. Nitrogen enrichment alters the resistance of a noninvasive alien plant species to Alternanthera philoxeroides invasion. Frontiers in Ecology and Evolution 11.

Wundrow EJ, Carrillo J, Gabler CA, Horn KC, Siemann E. 2012. Facilitation and Competition among Invasive Plants: A Field Experiment with Alligatorweed and Water Hyacinth. PLoS ONE 7.

Xu Q-Y, Wang D, Quan G-M, Xiang H-M, Zhang J-E. 2020. Invasive Chromolaena odorata species specifically affects growth of its co-occurring weeds. Annals of the New York Academy of Sciences 1470: 57-66.

Yang S, Ferrari MJ, Shea K. 2011. Pollinator Behavior Mediates Negative Interactions between Two Congeneric Invasive Plant Species. American naturalist 177: 110-118.

Yoshida K, Oka S. 2004. Invasion of Leucaena leucocephala and its effects on the native plant community in the Ogasawara (Bonin) Islands. Weed Technology 18: 1371-1375.

Zenni RD, da Cunha WL, Musso C, de Souza JV, Nardoto GB, Miranda HS. 2020. Synergistic impacts of co-occurring invasive grasses cause persistent effects in the soil-plant system after selective removal. Functional Ecology 34: 1102-1112.

Zhang X, Yu H, Lv T, Yang L, Liu C, Fan S, Yu D. 2021. Effects of different scenarios of temperature rise and biological control agents on interactions between two noxious invasive plants. Diversity and Distributions 27: 2300-2314.

Zhang X, Yu H, Yu H, Liu C, Fan S, Yu D. 2021. Highly competitive native aquatic species could suppress the growth of invasive aquatic species with similar traits. Biological Invasions 23: 267-280.

Zhang Z, Liu Y, Brunel C, van Kleunen M. 2020. Soil-microorganism-mediated invasional meltdown in plants. Nature Ecology & Evolution 4: 1612-1621.

Zhao C, Zhao X, Li J. 2022. Elevated CO2 and Increased N Intensify Competition between Two Invasive Annual Plants in China. Life-Basel 12.

Zhao C-Y, Liu Y-Y, Shi X-P, Wang Y-J. 2020. Effects of soil nutrient variability and competitor identify on growth and co-existence among invasive alien and native clonal plants. Environmental Pollution 261.

Zhi Y, Li H, An S, Zhao L, Zhou C, Deng Z. 2007. Inter-specific competition: Spartina alterniflora is replacing Spartina anglica in coastal China. Estuarine Coastal and Shelf Science 74: 437-448.

郭宇俊. 2024. 植物亲缘关系对共同入侵的影响研究. 博士, 沈阳农业大学.

何孟莹, 解青松, 李玥, 尹宣懿, 刘婷婷, 龙依依, 张震. 2024. 加拿大一枝黄花和小飞蓬共同入侵对植物群落的影响. 安徽农业大学学报 51: 359-367.

蒋智林. 2007. 入侵杂草紫茎泽兰与非入侵草本植物竞争的生理生态机理研究. 博士, 中国农业科学院.

刘云珊. 2024. 新发外来种对入侵种和本地种的影响. 硕士, 云南大学.

孟昀昊. 2022. 美洲商陆入侵的植物-土壤反馈机制探究. 硕士, 鲁东大学.

张瑞东. 2024. 紫茎泽兰和鬼针草入侵对滇池湖滨植物及土壤微生物群落结构的影响. 硕士, 云南大学.

张晓亮. 2020. 水生生物常见外来种的种间关系及其对乡土种的影响. 博士, 武汉大学.

赵林. 2007. 土壤营养对入侵杂草紫茎泽兰与替代植物黑麦草苗期竞争力的影响. 硕士, 南京农业大学.

朱宏伟. 2005. 替代植物黑麦草 *Lolium Perenne* 与入侵杂草紫茎泽兰 *Eupatoriuum adenophorum* 的竞争效应及其机制的研究. 硕士, 南京农业大学.

References for Supporting Information

Jones CG, Callaway RM. 2007. Invited Perspective. *Journal of Vegetation Science* **18**: 771-776.

Strauss SY. 1991. Indirect effects in community ecology: their definition, study and importance. *Trends in Ecology Evolution* **6**: 206-210.
